# Targeted single-cell RNA sequencing of transcription factors enhances the identification of cell types and trajectories

**DOI:** 10.1101/gr.273961.120

**Published:** 2021-06

**Authors:** Alexandra Pokhilko, Adam E. Handel, Fabiola Curion, Viola Volpato, Emma S. Whiteley, Sunniva Bøstrand, Sarah E. Newey, Colin J. Akerman, Caleb Webber, Michael B. Clark, Rory Bowden, M. Zameel Cader

**Affiliations:** 1 Translational Molecular Neuroscience Group, Weatherall Institute of Molecular Medicine, Nuffield Department of Clinical Neurosciences, University of Oxford, Oxford, OX3 9DS, United Kingdom;; 2 Wellcome Centre for Human Genetics, University of Oxford, Oxford, OX3 7BN, United Kingdom;; 3 UK Dementia Research Institute, Cardiff University, Cardiff, CF24 4HQ, United Kingdom;; 4 Department of Pharmacology, University of Oxford, Oxford, OX1 3QT, United Kingdom;; 5 Department of Psychiatry, Warneford Hospital, University of Oxford, Oxford, OX3 7JX, United Kingdom;; 6 Centre for Stem Cell Systems, Department of Anatomy and Neuroscience, The University of Melbourne, Parkville, Victoria 3010, Australia;; 7 The Walter and Eliza Hall Institute of Medical Research, Parkville, Victoria 3052, Australia;; 8 University of Melbourne, Department of Medical Biology, Parkville, Victoria 3052, Australia

## Abstract

Single-cell RNA sequencing (scRNA-seq) is a widely used method for identifying cell types and trajectories in biologically heterogeneous samples, but it is limited in its detection and quantification of lowly expressed genes. This results in missing important biological signals, such as the expression of key transcription factors (TFs) driving cellular differentiation. We show that targeted sequencing of ∼1000 TFs (scCapture-seq) in iPSC-derived neuronal cultures greatly improves the biological information garnered from scRNA-seq. Increased TF resolution enhanced cell type identification, developmental trajectories, and gene regulatory networks. This allowed us to resolve differences among neuronal populations, which were generated in two different laboratories using the same differentiation protocol. ScCapture-seq improved TF-gene regulatory network inference and thus identified divergent patterns of neurogenesis into either excitatory cortical neurons or inhibitory interneurons. Furthermore, scCapture-seq revealed a role for of retinoic acid signaling in the developmental divergence between these different neuronal populations. Our results show that TF targeting improves the characterization of human cellular models and allows identification of the essential differences between cellular populations, which would otherwise be missed in traditional scRNA-seq. scCapture-seq TF targeting represents a cost-effective enhancement of scRNA-seq, which could be broadly applied to improve scRNA-seq resolution.

Single-cell RNA-seq (scRNA-seq) is widely used to elucidate the biology of complex heterogeneous samples. However, scRNA-seq libraries commonly suffer from high dropouts (false zero expression estimates) and variability owing to both technical variation and the biological stochasticity of gene expression in individual cells (Kolodziejczyk et al. 2015). This means many scRNA-seq reads come from highly expressed genes that may not be informative for cell fate or function, while the biological information conveyed by other genes is lost. Low abundance essential genes, a class that includes many transcription factors (TFs), are particularly affected by the limitations of scRNA-seq (Lambert et al. 2018). TFs are prime regulators of gene expression that underpin much of cell biology from cell fate specification to disease processes. Therefore, a method to improve the detection and quantification of TFs in single cells would significantly increase the ability of scRNA-seq to address key biological and disease questions.

Human induced pluripotent stem cell (iPSC)-derived neuronal cultures are used in medical and research applications (Fernandez et al. 2013). However, high biological heterogeneity in developing neuronal cultures might hinder their widespread application (Volpato et al. 2018). To ensure reproducible generation of specific cell types from iPSC progenitors under the same protocol, it is crucially important to be able to compare differentiation outcomes within and between laboratories. We previously utilized scRNA-seq to examine the performance of an iPSC model of forebrain corticogenesis across multiple laboratories, identifying clear laboratory-dependent variability (Volpato et al. 2018). Therefore, we hypothesized in-depth single-cell profiling of TFs could allow us to decipher the differences in cell types and differentiation trajectories responsible for this variability.

We previously described RNA Capture-Seq, a method able to improve transcript detection and quantification in bulk samples with targeted RNA-seq (Mercer et al. 2014). Capture-Seq utilizes oligonucleotide probes targeted to gene or genomic regions of interest to enrich for expression from these regions, and it is especially useful when targeting lowly expressed transcripts (Clark et al. 2015; Bartonicek et al. 2017), even when samples are limiting (Curion et al. 2020). scRNA-seq enrichment methods to date have focused almost exclusively on the small family of highly expressed immune receptor genes, whereas Capture-Seq potentially provides the scalability and sensitivity required to be widely applicable (Riemondy et al. 2019; Saikia et al. 2019; Singh et al. 2019). Moreover, another potential advantage of Capture-Seq is a reduction in sequencing costs, because fewer reads are required per cell when sequencing is targeted to a small portion of the transcriptome. We now describe the application of a targeted approach to single-cell sequencing libraries, which we term scCapture-seq, using probes against 972 TFs (Curion et al. 2020). We applied our method to existing libraries generated from cultures of iPSC-derived cortical neurons, differentiated in two different laboratories using the same standard protocol (Volpato et al. 2018). We aimed to show (1) that scCapture-seq could overcome some of the limitations of scRNA by improving its sensitivity, and (2) that targeting TFs with scCapture-seq would uncover biology not visible with standard scRNA- seq by enhancing the identification of cell types and trajectories.

## Results

### Improved identification of transcription factors in single-cell RNA sequencing

**Figure 1.**
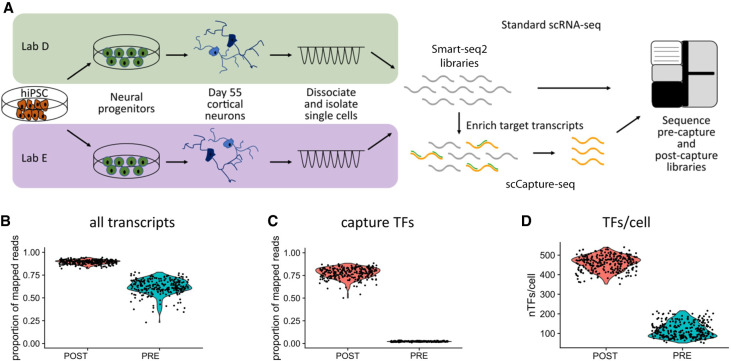
scCapture-seq improves mapping quality and identification of TFs. (*A*) Schematic of the experimental design. (hiPSC) Human induced pluripotent stem cell. (*B*,*C*) Post-capture (POST) increase in the proportion of reads assigned to all transcripts (*B*) and to the captured TFs (*C*) compared to pre-capture (PRE). (*D*) Increase in the number of TFs detected per cell post-capture.

Bulk RNA-seq and scRNA-seq was previously performed on iPSC-derived cortical neurons generated by two independent laboratories using identical cell lines and following the same established methods (referred to as Lab D and Lab E). This study revealed large variability in molecular phenotypes, highlighting factors underlying inter-lab variation. Cells for scRNA-seq were collected after 85 days differentiation and maturation in vitro (Volpato et al. 2018). We implemented TF capture on the single-cell libraries and analyzed 279 cells out of 376 captured cells that passed quality control (Methods; Fig. 1A). scCapture-seq resulted in an increase in the median number of mapped reads from 63.1% to 89.5% post-capture (Fig. 1B), whereas reads mapped to targeted TFs increased from 2.2% to 78.3% post-capture, resulting in a 36-fold enrichment for the transcripts of interest (Fig. 1C). There was consistent capture performance across all targeted genes (>10-fold enrichment) (Supplemental Fig. S1A). Target gene expression levels were on average increased ∼150-fold post-capture (Supplemental Fig. S1B) and highly correlated (*R* = 0.97) with pre-capture expression. In the pre-capture data, 585 of the targeted TFs were expressed with non-zero mean counts, but in post-capture there was a 25% increase in the number of TFs detected, for a total of 731 expressed TFs. Furthermore, the number of TFs detected in each cell increased more than fourfold (Fig. 1D). These results show excellent performance of scCapture-seq on single-cell libraries, allowing sensitive detection of the key transcriptional regulators.

### TF capture improves cell clustering and identification

**Figure 2.**
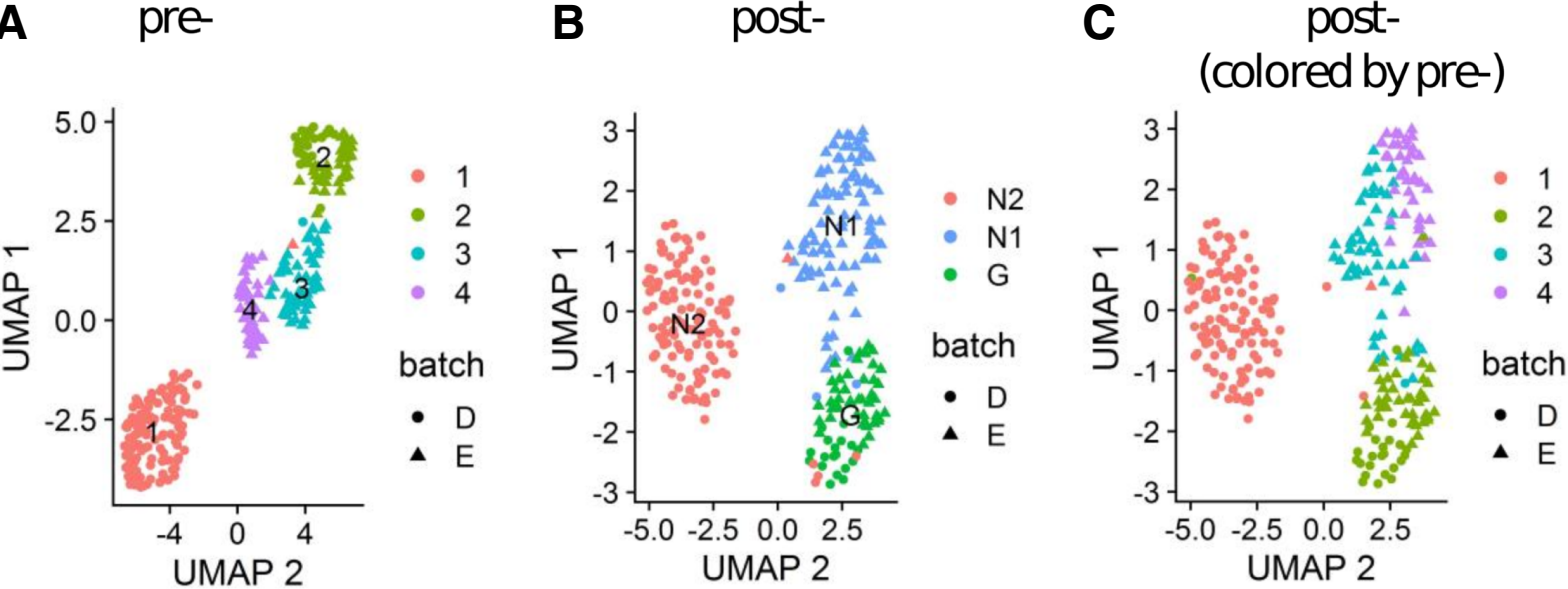
UMAPs of pre- and post-capture cells show distinct cellular clusters. UMAPs of pre-captured (*A*) and post-captured (*B*) clusters were constructed using the Seurat package. (*C*) Post-capture cells are colored by pre-capture clusters.

Clustering across 13,299 expressed genes with pre-capture data identified four populations of cells (Fig. 2A; Supplemental Fig. S2). Using markers of human brain cells (van de Leemput et al. 2014; Darmanis et al. 2015; Song et al. 2017), cluster 1 (Lab D) and clusters 3 and 4 (Lab E) were identified as neurons, and cluster 2 (mainly Lab E) as glial cells (Supplemental Fig. S2). As master transcriptional regulators, clustering by TFs (which in scRNA-seq data sets are underrepresented owing to generally low expression) might improve assignments of cell identity. Clustering based on both pre-capture and post-capture TF expression (585 vs. 731 expressed TFs) divided cells into three groups, which we termed neuronal-like 1 (N1), neuronal-like 2 (N2), and glial-like (G) (Fig. 2B; Supplemental Fig. S3A,B). The capture improves the clustering by creating a clearer distinction between the clusters (Supplemental Fig. S3A,B) and increasing the number of differentially expressed TFs from 129 to 155 post-capture (Supplemental Fig. S3D,E). The neurons from Lab E (pre-capture clusters 3 and 4) coalesced into a single post-capture cluster N1 (Fig. 2C). This merge is consistent with the differential expression of only 4 TFs (*BHLHE22*, *NFIX*, *ZBTB20*, and *NHLH1*) between pre-capture clusters 3 and 4 and the observed similarity between these clusters pre-capture (Fig. 2A; Supplemental Fig. S2A,C).

TF capture improved the precision of hierarchical clustering (Supplemental Fig. S3), increasing the number of differentially expressed genes (DEGs) in the N2 cluster (Lab D) from nine pre-capture to 53 post-capture. In addition, partial reassignment of G cells from Lab E to the N1 cluster (Supplemental Fig. S3A,C) increases the number of post-capture N1 DEGs, and decreases the number of DEGs in cluster G (Supplemental Fig. S3D,E). In further support of the biological value of the capture, we next analyzed the expression of 107 key TFs involved at early stages of neuronal differentiation (Inoue et al. 2019). We found that twice as many cells expressed key neural induction TFs post-capture; moreover, there was more homogenous expression of critical TFs in each cluster (Supplemental Fig. S4).

Closer inspection of TF DEGs revealed that cells of the glial-like cell cluster G express TFs that included *HES1*, *HES5*, *PAX6*, *NR2E1*, *TCF7L2*, *OTX1*, as well as *GFAP*, consistent with a radial glial or proliferative neural progenitor identity (Supplemental Fig. S5; Darmanis et al. 2015; Telley et al. 2016). This was unexpected, because a reliance on the standard cell identity markers suggested an astrocytic glial identity as previously reported (Volpato et al. 2018). Neuronal-like N1 cells expressed neuronal TFs such as *NEUROD2*, *TBR1*, *NEUROG2*, *EOMES* (also known as *TBR2*), *MYT1L*, and *BHLHE22*, consistent with the development of deep layer excitatory cortical neurons. In contrast, neuronal-like N2 cells expressed a distinct set of TFs, which included *MEIS1*, *SP9*, *DLX2*, and *DLX6*, signifying an interneuron identity (Lim et al. 2018). Hence TF-based cell identity classification using capture sequencing data uncovered unappreciated details about the cell types present in the iPSC neuronal cultures. Specifically, an apparent glial cell cluster was in fact a neural progenitor population, and neuronal cell clusters from two laboratories were distinguished as excitatory cortical neurons and inhibitory interneurons. It is therefore likely that different differentiation trajectories in Labs D and E were the principal cause of variation in phenotypic outcomes for the experiment described by Volpato et al. (2018) rather than differences between neuron and glial ratios as had been concluded.

**Figure 3.**
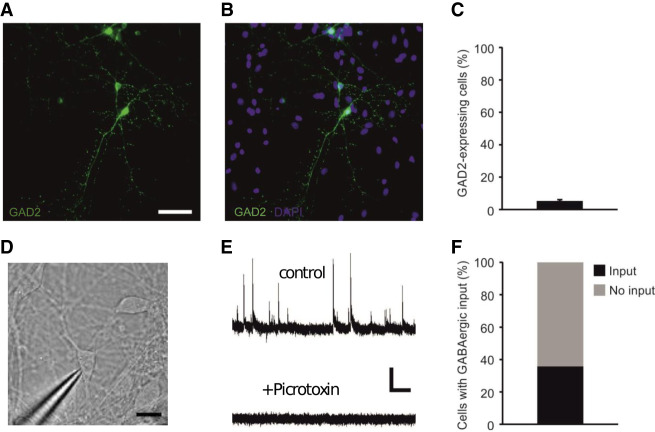
TF scCapture-seq observations are corroborated by immunostaining and cell physiology. (*A*,*B*) A subpopulation of iPSC-derived cortical neurons were immunopositive for the inhibitory interneuron marker, GAD2, and represented a small subset of DAPI-positive nuclei. (*C*) Population data showing proportion of GAD2-positive cells (*n* = 12 fields of view [FOVs] from three differentiations). (*D*) iPSC-derived cortical neurons were targeted for whole-cell patch clamp recordings. (*E*) A subset of neurons showed spontaneous outward synaptic currents, consistent with the presynaptic release of GABA from interneurons (*top*), which could be blocked with the GABA A receptor antagonist, picrotoxin (100 μM; *bottom*). (*F*) Population data showing the proportion of neurons receiving GABAergic synaptic input (*n* = 14).

The generation of GABAergic interneurons from a cortical differentiation protocol that has been validated to produce glutamatergic excitatory neurons and astrocytes was not expected (Shi et al. 2012a,b); however, it was potentially consistent with recent findings (Strano et al. 2020). Therefore, to corroborate our transcriptomic observations, we performed immunohistochemistry and patch clamp electrophysiology on neurons differentiated in Lab D. In accordance with our scCapture-seq findings, these cultures contained GAD2 (also known as GAD65) immunopositive neurons (Fig. 3A–C) and showed functional GABAergic synaptic connections (Fig. 3D–F). Our data further support the validity of using TF targeting for improved classification of neuronal populations.

### TF capture improves the resolution of gene regulatory networks

**Figure 4.**
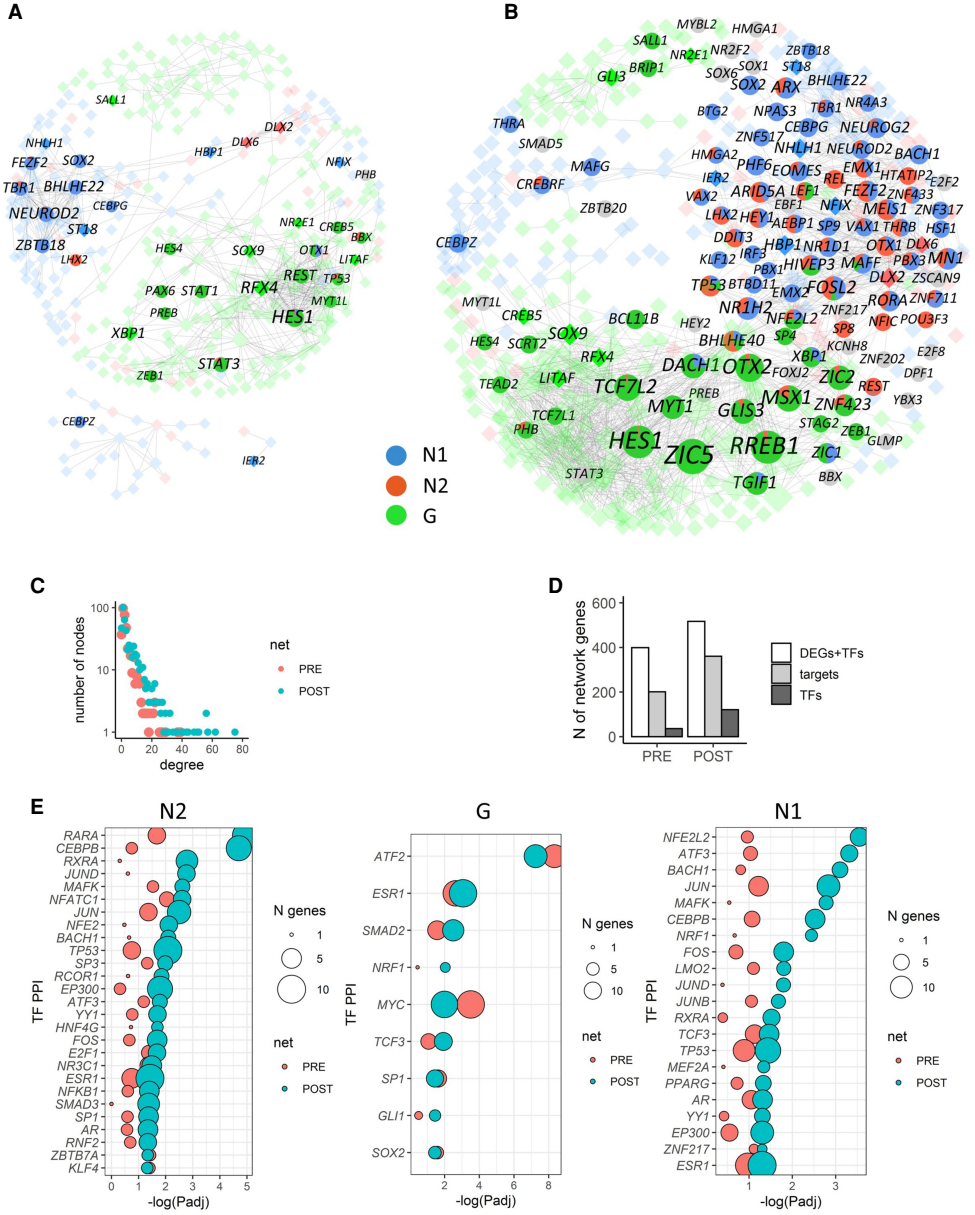
TF scCapture-seq improves gene regulatory networks. The networks of coexpressed pre-capture genes with (*B*) or without (*A*) imputed post-capture TFs. Only genes that are differentially expressed between three cell groups are shown. The TF nodes are labeled, with node sizes reflecting their degree on the whole network. TF nodes that are differentially expressed post-capture are shown as embedded pie charts, with colors corresponding to the proportion of DEGs neighbors from each of the three cell types. TFs that are not connected to cluster DEGs are gray. TFs that are also pre-capture DEGs are shown by brighter colored squares. (*C*) Distribution of network degree between nodes in pre-capture and post-capture imputed networks. (*D*) Total numbers of genes (DEGs + TFs), TFs, and TF targets present on the pre-capture and post-capture-imputed networks. (*E*) Enrichment of the cluster subnetworks in TF PPIs (TF networks), for pre-capture (PRE) and imputed (POST) networks. The circle sizes show the number of genes in each TF PPI term.

To investigate whether our TF scCapture-seq might enhance TF-gene networks underpinning the cell clusters, we built a coregulation network of coexpressed genes using the whole transcriptome. Including imputation of post-capture TF expression, rescaled according to the differences in library sizes, resulted in improved resolution of the gene regulatory network (GRN), which contained 1.5 times more nodes and two times more edges (Methods). We also constructed a network focusing solely on DEGs between clusters. The pre-capture GRN (Fig. 4A) had poor representation of N2 cluster genes, and N1 cluster genes were disconnected from the main network. Utilizing the post-capture information, the imputed GRN had 517 nodes (121 TFs) and 1939 edges (Fig. 4B), with much improved N2 cluster representation (47 nodes, 591 edges); whereas the N1 cluster genes were now integrated inside the network. Both pre-capture and imputed GRNs were enriched in the targeted TFs (*P* < 2 × 10^−7^, calculated using network enrichment analysis [NEA]) (Alexeyenko et al. 2012). However, the imputed network identified substantially more connections between genes, greatly increasing network degrees (numbers of genes connected to each node), in particular for the hub genes (Fig. 4C). Furthermore, the number of TF targets present in the GRNs was significantly increased, from 50% pre-capture to 70% post-capture (Fisher's exact test, *P* = 4 × 10^−9^) (Fig. 4D; Supplemental Table S1), suggesting that newly identified TFs post-capture are functionally related to DEGs between clusters. Moreover, our analysis of known protein–protein interactions (PPIs) between TFs and other proteins (Kuleshov et al. 2016) revealed that TF PPIs links, which are present in the GRNs, were significantly enriched in the imputed post-capture GRN clusters (*P*-value of exact Wilcoxon-signed-rank test for the effect size is 1.9 × 10^−6^) (Fig. 4E; Supplemental Table S2). Overall, incorporation of the capture data improved the resolution of the GRNs and enabled mechanistic insights into TF-mediated biological networks.

### TF capture facilitates the identification of TFs associated with developmental trajectories

**Figure 5.**
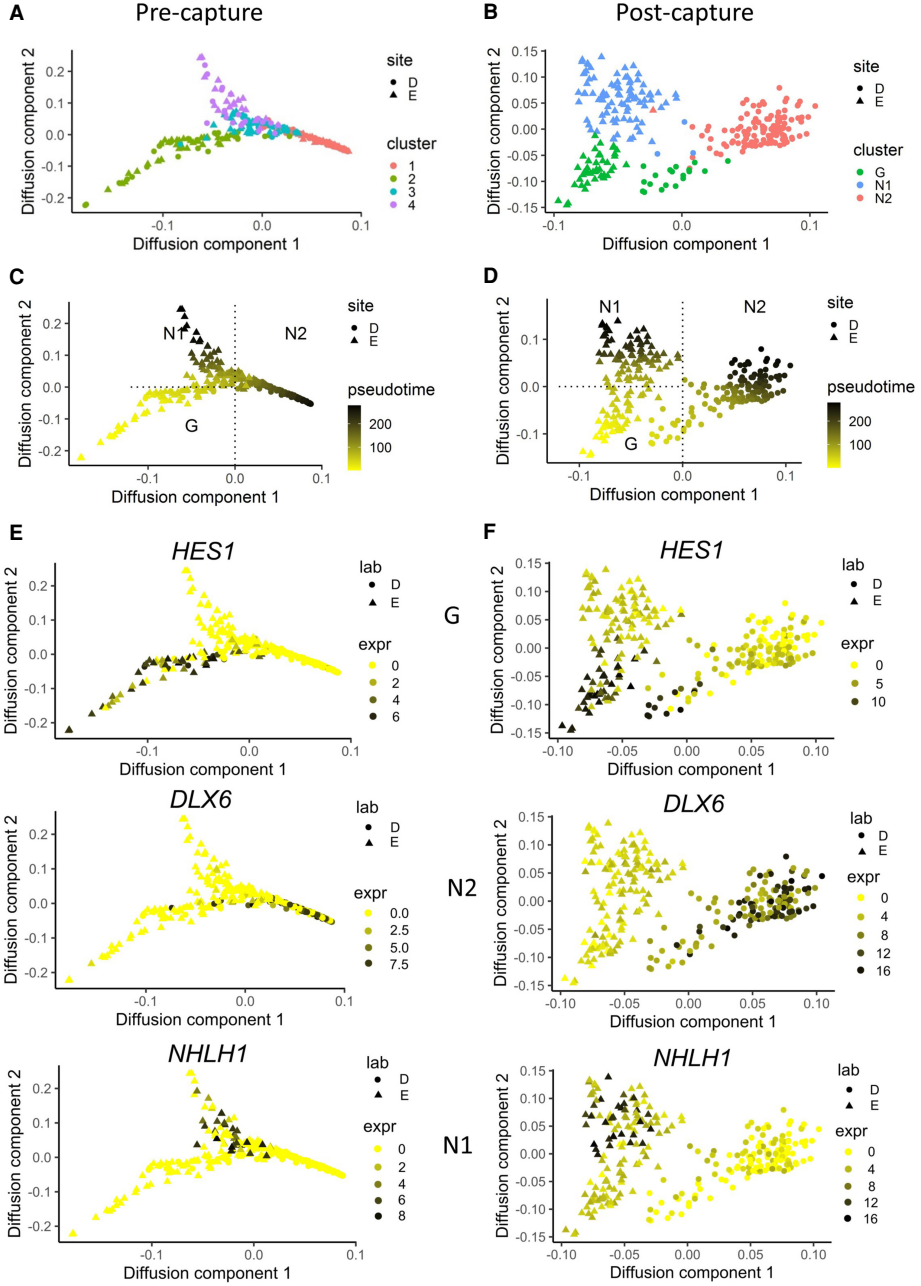
TF scCapture-seq improves analysis of cell differentiation trajectories. Diffusion maps of pre-capture (*A*,*C*,*E*) and post-capture (*B*,*D*,*F*) cells built using the *destiny* package (Angerer et al. 2016) with pseudotime direction determined using Slingshot (Street et al. 2018). The cells are colored by clusters (*A*,*B*) or pseudotime (*C*,*D*). (*E*,*F*) Log_2_ expression of representative genes for each cell group on pseudotime space for pre-capture (*E*) and post-capture (*F*).

To explore the differentiation trajectories and TFs involved in cell fate determination in each cluster, we ordered cells in pseudotime (Angerer et al. 2016). The expression profiles of TFs with known maturation kinetics (Telley et al. 2016) were used to establish a pseudotime direction (Fig. 5A–D; Supplemental Fig. S5), in which cells of the G cluster occupied an earlier position, consistent with their neural progenitor identity (Darmanis et al. 2015; Telley et al. 2016). Using either pre- or post-capture genes resulted in a qualitatvely similar ordering of cells (with G cells being the earliest on pseudotime, followed by two later branches of N1 and N2 cells), but the precision of expression change estimates for individual genes was greatly improved post-capture (Fig. 5E,F; Supplemental Fig. S5). This was manifested by the increased numbers of cells with detected TFs. For example, the number of cells expressing HES1 increased from 56 to 224 post-capture (Fig. 5E,F).

**Figure 6.**
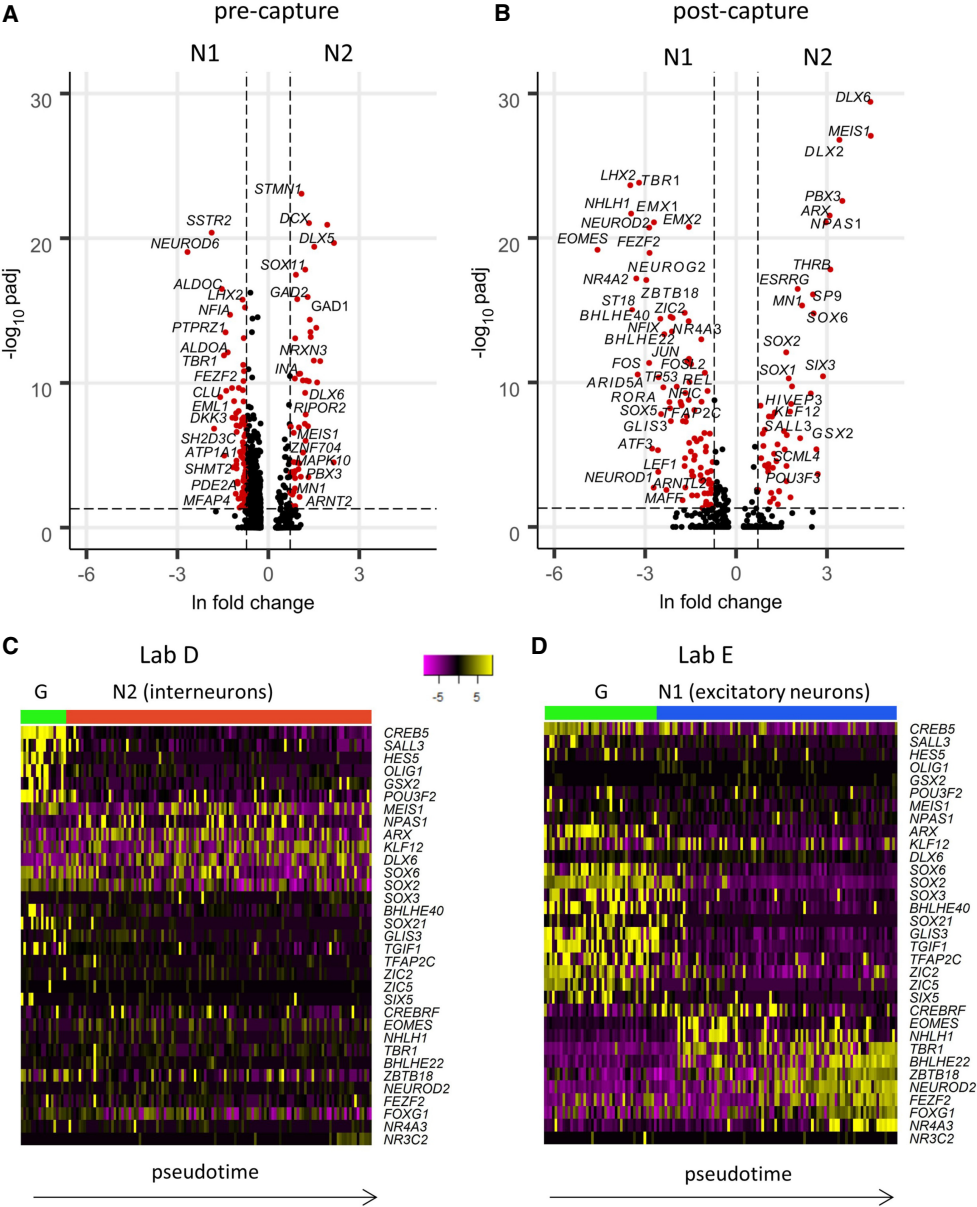
TF scCapture-seq reveals differentially expressed genes underlying inter-laboratory variability. (*A*) Volcano plot of all pre-capture DEGs between N1 and N2 neurons (based on pre-capture gene expression in cells of cluster 1 vs. cells of clusters 3 and 4). (*B*) Volcano plot of all post-capture DEGs between N1 and N2 neurons (based on post-capture gene expression in cells of clusters N1 vs. N2). Significant DEGs are labeled (FDR ≤ 0.05, natural log fold changes ≥ 0.5). (*C*,*D*) Heat maps of post-capture expression of the key TF DEGs with different temporal kinetics between the laboratories. Each heatmap includes only cells from the respective laboratory: Lab D (*C*) or Lab E (*D*). The cells are ordered by pseudotime (based on all cells, as in Fig. 5A,B), including cells of the earlier G cluster. Expression is log_2_-normalized and centered.

Pseudotime ordering revealed distinct developmental trajectories for neurons from Lab D (N2) and Lab E (N1). Also, TF capture, but not pre-capture data, distinguished the G cells from the two laboratories, suggesting that a common progenitor state was not represented in the cultures at the point of harvest (Fig. 5C,D). To identify the set of TFs that might be involved in the developmental switch between the two neuronal subpopulations, we performed a differential expression between the respective clusters of N1 and N2 neurons (Fig. 6A,B; Supplemental Fig. S6A), or between G cells from different laboratories (Supplemental Fig. S6B). One hundred sixty-one TFs were differentially expressed between N1 and N2 post-capture (Fig. 6B), compared to only 65 TFs pre-capture (Supplemental Fig. S6A). Among these post-capture exclusive DEGs, there were multiple TFs whose kinetics over pseudotime differed between the laboratories (*SALL3*, *GSX2*, *BHLHE40*, *SOX3*, *TFAP2C*, *GLIS3*, *POU3F2*, *SP4*, *CREBRF*) (Fig. 6C,D). The post-capture expression of laboratory-specific TFs revealed different trends in pseudotemporal kinetics in two laboratories. Lab E cells showed a clear temporal ordering of TF expression, recapitulating the expected progression during development. Initially many neural progenitor genes were strongly expressed in Lab E cells before their expression was extinguished and cells instead successively expressed cortical excitatory neuron TFs such as *EOMES*, *TBR1*, and *FOXG1*. In contrast, fewer Lab D cells expressed neural progenitor markers, and the N2 neurons had less pronounced pseudotemporal patterns, suggesting a more uniform, mature developmental state. The G cells from Lab D strongly expressed *HES5* and *SALL3*, whereas Lab D neurons expressed *DLX2*, *DLX6*, and *SP9*, which define a particular interneuron program (Lim et al. 2018).

### TF capture identifies retinoic acid signaling as a candidate mechanism driving differences in developmental trajectories

We hypothesized that scCapture-seq may reveal previously unappreciated details of how a differentiation process could generate variability when the same protocol is applied to the same cell line. Indeed, the TF PPIs of the N2 cells showed significant evidence for retinoic acid (RA) signaling (*RARA*, *RXRA*, and *CEBPB*) that was not apparent with pre-capture data alone (FDR = 1 × 10^−5^ post-capture vs. 0.02 pre-capture for *RARA*) (Fig. 4E). We therefore used the post-capture imputed GRN from Figure 4B to explore the potential RA-related effects. The RA subnetwork highlighted a highly interconnected network, with multiple direct connections between RA TFs (e.g., *RORA*, *NR1H2*, *ZNF423*) and N2-specific GABAergic (interneuron) genes such as *GAD1*, *GAD2*, and *SLC6A1*, as well as interneuron TFs such as *DLX2* and *DLX6* (Supplemental Fig. S7A). The RA TFs were negatively correlated to the interneuron TFs, consistent with the down-regulation of RA TFs in N2 neurons (Supplemental Fig. S7B) and available evidence on the induction of interneuronal genes *MEIS* and *DLX* by the abrogation of RA signaling (Wahl et al. 2018). Together, these observations identify RA signaling as a candidate mechanism, potentially mediating the inter-laboratory differences in cell fate choices.

In summary, these data establish that scCapture-seq targeting of TFs can improve the sensitivity for resolving cell type differences among neuronal populations and identify the changes in gene regulatory networks that underlie these differences.

### Validation of scCapture-seq approach with an independent microfluidics-based scRNA-seq sample

To further validate the scCapture-seq approach, we applied it to an independent pool of scRNA-seq libraries made using the Fluidigm C1 microfluidics and Smarter-seq protocol. This sample of intestinal stromal cells from ulcerative colitis (UC) patients has been previously described and is characterized by the expression of inflammatory genes (Kinchen et al. 2018). We performed scCapture-seq with our TF panel on 126 out of 191 single-cell libraries that passed quality control (Methods). TF expression levels in post-capture libraries were on average increased 60-fold compared to pre-capture libraries, with a high correlation (*R* = 0.91) between pre- and post-capture expression (Supplemental Fig. S8A). All the targeted TFs were enriched post-capture (>10-fold) (Supplemental Fig. S8B), leading to an approximately threefold increase in the number of captured TFs per cell (Supplemental Fig. S8C). We next clustered the pre-capture cells using all genes with non-zero mean counts, which revealed two distinct clusters among the 125 cells (Supplemental Fig. S8D). Two very similar clusters were observed post-capture (Supplemental Fig. S8D), further confirming that using post-capture libraries expressing only the targeted TFs is sufficient to characterize cellular populations. Moreover, we found that several TFs that are expected to be involved in intestinal inflammatory processes, such as *TCF7L2*, *PBX1*, *TCF21*, and *FOXO3* (Chawla et al. 2013; Kim et al. 2017; Crow and Gillis 2018), were differentially expressed between the two clusters post-capture, but not pre-capture (Supplemental Table S4; Supplemental Fig. S8E,F). Overall, using an independent scRNA-seq data set we confirmed that scCapture-seq greatly improved the identification of the targeted TFs, potentially facilitating the discovery of important regulatory TFs underlying the differences between cellular populations. These results also establish that scCapture-seq works robustly on both whole-transcript plate-based and 3′-end microfluidics-based sample libraries, although further optimization for non-Smart-seq library types would be possible (see Discussion).

### Validation of scCapture-seq with a separate target gene panel

We next validated the scCapture-seq approach with the NeuroGWAS (NG) panel (Curion et al. 2020), whose target genes include markers for neuronal development and identity and candidate risk genes for schizophrenia and Parkinson's disease. We analyzed 359 out of 376 captured iPSC-derived cells from cortical neuron cultures (Volpato et al. 2018), which passed quality control. Eighty-seven out of 101 NG genes were expressed post-capture. Similar to TF capture, there was a linear correlation between pre- and post-capture expression: >10-fold enrichment of NG genes and a 3.8-fold increase in the number of captured NG genes per cell (Supplemental Fig. S9A–C). Moreover, clustering identified five similar cellular communities in pre- and post-capture data (Supplemental Fig. S9D), confirming the validity of using post-capture for cell type identification. Using the cell type markers we confirmed that, similar to TF capture cultures, NG capture cultures were represented by neuronal cells from Lab D (clusters 1 and 2) and Lab E (clusters 3 and 4), as well as glial-type cells (cluster 5) (Supplemental Fig. S10). Differential expression of NG genes revealed that most of the candidate disease genes, for example, *BAG3*, *RAB29, STX4*, and *FAM126A*, potentially associated with Parkinson's disease; and *CACNB2*, *EMX1*, and *AKT3* (potentially associated with schizophrenia) had higher expression in Lab E neurons and/or glial cells, with only *HIP1R* having higher expression in Lab D neurons (Supplemental Fig. S9E,F). One partial reason for these observations could be the less mature, more developing state of Lab E neurons. These results show that scCapture-seq can also be successfully applied to other sets of capture pools, including NG genes, to facilitate the identification of the disease genes in different cell types.

## Discussion

We applied targeted sequencing to a large set of biologically critical genes that are typically poorly represented in single-cell data to investigate if this method can enhance the biological information discoverable using scRNA-seq. As a proof of principle, we targeted most known human TFs (approximately 1000) and applied our approach to iPSC-derived neurons. We found that capture was highly effective at recovering TF single-cell gene expression. Compared to pre-capture data, we observed a 36-fold enrichment for TF reads, increasing the total number of TFs detected in the sample, and each cell was shown to express a broader range of TFs. The high correlation between post-capture and pre-capture expression suggested there was little bias in relative TF expression levels attributed to capture. Many of the key TFs were poorly represented pre-capture, precluding the downstream analysis of the neuronal fate specification based on pre-capture data alone. Our results show that performing TF scCapture-seq greatly improved our understanding of the underlying biology in the system, and when combined with standard full transcriptome scRNA-seq, allowed at low additional cost the construction of a comprehensive gene regulatory network and additional insights into the processes driving neuronal differentiation. We also validated our approach on a different scRNA-seq data set of intestinal stromal cells prepared using the Fluidigm C1 microfluidics and Smarter-seq approach and on a different panel targeting neurological disease genes. In both cases we confirmed the enrichment of the targeted genes and the potential for scCapture-seq to uncover new biological insights.

Previous application of RNA Capture to bulk and low input samples has shown that this method improves sequencing sensitivity, allowing the detection and quantification of transcripts that are poorly represented in standard libraries (Mercer et al. 2014; Clark et al. 2015; Curion et al. 2020). Here, we show capture has similar advantages when applied to single cells, which are well known to have low sensitivity and a high noise threshold. Therefore, the benefit sequence capture provides to biological analyses of single cells may be greater than for bulk samples. Previous targeted single-cell methods have shown the enrichment of individual cells of interest by PCR for specific cell barcodes (Ranu et al. 2019) or immune receptor genes using LNA or DNA capture probes (Riemondy et al. 2019; Singh et al. 2019). Although these studies showed that an improved biological understanding could be obtained through targeted single-cell sequencing, the number of targeted cells or genes was very limited. PCR and LNA-probe-based methods have practical and cost limitations for scaling to a large number of targets, whereas targeting highly expressed immune receptor genes left the applicability of single-cell capture to lowly expressed target unresolved. Our results now show the utility of applying single-cell capture to a large numbers of genes, including those with low expression levels.

ScCapture-seq was primarily designed for Smart-seq2 libraries. We have demonstrated that scCapture-seq is compatible with libraries generated by Fluidigm C1 microfluidics and Smarter-seq. We have not yet evaluated scCapture-seq on droplet-based single cell libraries but believe our method can be adapted to these types of libraries. In this case, we recommend utilizing adaptor blocking oligonucleotides specifically designed for compatibility with the barcode and index features of each methodology. Additionally, for those single-cell techniques that produce transcript counting (e.g., 10x Genomics, MARS-seq, etc.) rather than whole-transcript libraries, capture probe designs could be optimized to cover only those 3′ or 5′ regions likely to be sequenced (Supplemental Fig. S11). One important difference between scCapture-seq and 10x Genomics Targeted Gene Expression is that our method can be applied to a range of single-cell methodologies and library types. It can provide similar advantages to the 10x targeted gene expression approach but is also compatible with methods like Smart-seq that preserve information across the full-length of transcripts and so potentially can be used for the identification of splice isoforms in a way that is not possible using 3′ or 5′ 10x Genomics Targeted Gene Expression measurements. Thus, TF capture resulted in a median threefold increase in detected splice junctions in our data set, similar to what was previously reported (Curion et al. 2020). scCapture-seq can be used as a cost-effective substitution for standard scRNA-seq, especially for larger projects, or multiple projects for which the same capture design can be used. Although cost savings depend on many variables, including local costs and the number of additional samples able to be combined in a sequencing lane, we find Capture-Seq enables 5–10 times less sequencing to be performed and saves money once five lanes of sequencing have been avoided. One important limitation to all single-cell enrichment methods is that they can only enrich for genes present in the sequencing library and cannot overcome the inefficiencies of single-cell library generation. As single-cell library method efficiencies continue to improve (Hagemann-Jensen et al. 2020), the sensitivity of single-cell capture will progressively increase.

The differentiation of iPSCs into defined neuronal populations provides an excellent opportunity to understand developmental processes, because these cultures typically contain a diversity of cell subtypes and different stages of maturation. Protocols developed to produce forebrain cortical excitatory neurons are now well established and widely used. Typically, they involve dual SMAD inhibition to induce a neural ectoderm fate followed by maturation into a default forebrain cortical neuronal specification (Picelli et al. 2013). scRNA-seq has proved to be an excellent tool to characterize the heterogeneity of cultures, but without access to critical sets of genes, namely TFs, the potential to reveal mechanistic processes is significantly hampered. The data obtained from our TF capture highlights the importance of TF data in understanding cell biology and revealed unexpected differences in developmental programs arising from an identical differentiation protocol conducted on the same donor iPSC lines in two different laboratories. We found that Lab E neurons expressed TFs involved in the development of cortical excitatory neurons, whereas Lab D neurons expressed inhibitory interneuron TFs. This was unexpected, because the protocol used does not include any factors to direct cells to an interneuron fate. We found that Lab D culture differentiate into a particular type of immature interneurons, expressing *DLX* (*DLX2*, *DLX6*, *DLX1*) and *SP* (*SP8*, *SP9*, *SP4*) TFs (Lim et al. 2018). Our results are in agreement with recent findings that cortical neuron specification from iPSCs is strongly modified by the fluctuations of factors affecting regional patterning of the brain, including RA levels and the activity of Wnt signaling pathway (Strano et al. 2020). For example, it was shown that insufficiency of Wnt signaling is capable of switching the culture from the formation of dorsal excitatory neurons to ventral inhibitory interneurons, particularly to the highly ventralized NKX2-1 type of interneurons (Strano et al. 2020). We found that Lab D culture differentiations included *DLX/SP* expressing interneurons, which are distinct from NKX2-1-dependent interneurons (Lim et al. 2018), and may be less influenced by Wnt signaling (Strano et al. 2020). Other RA-mediated effects may therefore be more relevant for cell fate choices in our experiment.

To investigate the potential mechanism underlying the different development trajectories of neurons from different laboratories, we used the post-capture-inferred coexpression networks. We found that the interneuron subnetwork was significantly enriched in genes of retinoic acid (RA) signaling (e.g., *RORA*, *NR1H2*, and *ZNF423*). The RA genes were mainly down-regulated in these neurons, but highly connected and anti-correlated to the interneuronal genes (e.g., *DLX2* and *GAD1*), suggesting that interneuronal specialization is related to the suppression of the RA pathway in Lab D cultures. Reduction in RA signaling was previously reported to up-regulate key interneuronal TFs of the *DLX* family (Wahl et al. 2018). RA is also known to be an important factor in neuronal differentiation, having complex and concentration-dependent effects (Crandall et al. 2011). A potential explanation for the reduced RA signaling in Lab D compared to Lab E is differences in culture growth. Our previous work showed that passage number before differentiation, media volume changes, and other factors contributed to the variability between laboratories, potentially affecting culture growth and differentiation rates (Volpato et al. 2018). In addition, our pseudotime analysis suggests that Lab E cells are actively developing, but Lab D cells are more stable. Therefore, it is possible that faster initial growth and development of Lab D neurons caused the depletion of RA and altered cell fates. Overall, our analysis suggests that careful control of RA concentration during iPSC differentiation into cortical neurons might improve the reproducibility of the differentiation protocol.

To conclude, we showed that targeted TF sequencing, scCapture-seq, greatly improves the resolution of biological information derived from scRNA-seq. scCapture-seq alone can be used as a cost-effective substitution for standard scRNA-seq, improving cell type characterization and the analysis of developmental trajectories. We applied scCapture-seq to previously published data on iPSC-derived neurons and showed that, because of greatly improved TF detection, scCapture-seq enabled the identification of key developmental TFs driving cellular heterogeneity. We subsequently showed that combining TF scCapture-seq with standard, whole-transcriptome scRNA-seq recovered more comprehensive gene regulatory networks than scRNA-seq alone, in this case implicating retinoic acid signaling as a key factor in cellular heterogeneity. Our approach has widespread application, because correct detection of TF expression in single cells will enable improved identification of cell types, trajectories, and GRNs present during development, physiological or pathological states, or in response to drug perturbations. ScCapture-seq could also be adapted to capture other potentially interesting genes using bespoke oligonucleotide probes. Our approach could be immediately applied to any existing Smart-seq2 single-cell RNA-seq library to cost-effectively enhance the biological insights available with scRNA-seq.

## Methods

We resequenced our previous scRNA-seq libraries (Volpato et al. 2018), using targeted sequencing (scCapture-seq) for 972 known human TFs, as previously described (Curion et al. 2020). Briefly, the cell cultures were differentiated from human iPSCs derived from dermal fibroblasts using the dual SMAD inhibition protocol and collected on day 55 after plating. A potential source of vitamin A for endogenous RA synthesis in the cells of these cultures was a media B27 supplement (Volpato et al. 2018). Libraries were prepared from 376 day 55 neurons (and eight “mini-bulk” samples) using the Smart-seq2 protocol (Picelli et al. 2013) and then used for both pre- and post-capture sequencing. Each sample was spiked with the equivalent of 1 μL of a 1:10,000,000 dilution of the ERCC RNA Spike-In Mix 1 (Thermo Fisher Scientific). Libraries were pooled at 384-plex, and each pool was sequenced on one lane of a HiSeq 4000 (75-base paired end reads). The average size of post-capture libraries were four times larger than the size of pre-capture libraries. The cells were from two different laboratories (named D and E in Volpato et al. 2018), and our analysis confirmed the previous observation of the differences between the cell populations originating from different laboratories (batches). Because the cells from different laboratories showed substantial differences, we did not correct for the batch effect in our analysis.

### TF capture

The oligonucleotide capture probes were designed to target 972 human DNA-binding TF genes (Curion et al. 2020). These high-confidence TFs were previously chosen based on the Tfcheckpoint database (Chawla et al. 2013; http://tfcheckpoint.org), using a manually curated list of sequence-specific DNA-binding transcription factors (DbTF). Although the TF capture design could be extended to include additional recently identified TFs, it is important to be mindful of the proportion of the transcriptome captured, because this affects the enrichment. Our previous modeling of the effect of the capture pool size on enrichment suggested that the chosen set of approximately 1000 TFs enables successful enrichment, while targeting a large number of TFs that represents the biological diversity in human cells (Curion et al. 2020). Probes were also present for 42 abundant TFs, targeted in part, and 221 control brain-specific genes, which were not used in the present analysis, as well as sets of synthetic control genes including SIRVs, Sequins, and a partial set of the ERCC spikes-in, comprising 56 of the 92 ERCCs. Target regions were trimmed to remove any potential unwanted overlap with non-target genes, highly expressed RNA repeats, or pseudogenes. The probe design was previously fully described (Mercer et al. 2014; Curion et al. 2020). Probe synthesis was performed by Roche NimbleGen. Target capture was performed as previously described (Curion et al. 2020) using the protocol from Mercer et al. (2014) with slight modifications. Briefly, 850 ng of pooled Smart-seq2 libraries were used for capture hybridization along with Cot1 and blocking oligos (xGen NXT Universal Blockers, IDT). Hybridization was performed for 3 d. Post-capture LMPCR was performed for 12 cycles per the SeqCap RNA Enrichment System User's Guide V1.0 (Roche) with KAPA Taq and Roche post-cap LMPCR primers, except that PCR input was 17 µL of resuspended capture beads. QC of captured libraries was performed by Qubit (Thermo Fisher Scientific) and TapeStation (Agilent) to measure post-capture library concentration, yield, and size distribution. Successful capture enrichment was confirmed by qPCR (QuantStudio 6, Thermo Fisher Scientific). Libraries were sequenced at the Wellcome Trust Centre for Human Genetics (WTCHG).

### scRNA-seq analysis

The scRNA-seq libraries were analyzed similarly to Volpato et al. (2018), except that we used more recent reference annotations: hg38.p10 with GENCODE Release 26 annotation. The average size of pre-capture libraries was 1.5 million read pairs. The contamination with ribosomal rRNA was removed with the Sortmerna package (Kopylova et al. 2012). The reads were mapped to the hg38 genome using the STAR aligner (Dobin et al. 2013) as before. The average proportion of uniquely mapped reads was 59%. Gene counts for the mapped reads were obtained using the FeatureCounts function of the Subread package (Liao et al. 2013). For the analysis we retained only genes expressed with mean counts > 1 and filtered out the low-quality cells, based on pre-capture libraries, as before (Volpato et al. 2018). Briefly, we filtered out the cells expressing fewer than 2000 genes, with an initial library size less than 0.5 million of mapped read pairs, having low complexity (200 most expressed genes representing more than 50% of all counts), and cells with low endogenous RNA (ERCC spikes-in representing more than 14% of all counts). Two hundred seventy-nine cells passed quality control and were used in the analysis. The proportion of reads assigned to the capture TFs was calculated as the sum of TF-mapped counts divided by library size. Increasing the expression threshold for gene expression (e.g., to mean counts 4) preserves the post-capture TF enrichment. Post-capture genewise enrichment was calculated using CPM normalized counts. For each gene, the enrichment was a ratio between the average CPMs in the post- and pre-capture libraries, plotted in log_2_ scale by adding a pseudocount of 1.

### Fluidigm C1 microfluidics library capture

To validate scCapture-seq using a different data set, we also captured our TF panel in 126 out of 191 intestinal stromal cells from UC patients, which passed the quality control. The scRNA-seq was made using the Fluidigm C1 microfluidics and Smarter-seq protocol (Kinchen et al. 2018). TF capture was performed, and the libraries processed as described above. Post-capture libraries were sequenced on one lane of a HiSeq 4000 (75-base paired end reads). The total number of the targeted TFs, which were expressed with non-zero mean counts, was increased post-capture from 530 to 631 TFs.

### NeuroGWAS capture

To validate scCapture-seq on a different capture panel, we performed capture using the previously designed NeuroGWAS (NG) panel targeting transcripts implicated in neurological diseases and traits (Curion et al. 2020). The NG capture was performed on Smart-seq2 libraries from a separate pool of 376 iPSC-derived cortical neurons compared to the TF capture. Capture hybridization and sequencing was performed per the TF cortical neuron capture. Out of 376 cells, 359 passed the quality control and libraries were processed as described above. Out of 101 NG genes, 71 genes were expressed pre-capture, and 87 genes were expressed post-capture.

### Clustering and differential expression analysis

Most of the initial steps of the analysis, including clustering and differential expression were done in the Seurat version 3 package (Stuart et al. 2019). Heat maps were based on differential expression (FDR ≤ 0.05) in each cluster against all other cells. Volcano plots were based on differential expression between the N1 and N2 neurons or between G cells from Lab D and Lab E. Pre-capture N1 and N2 neurons were defined as cells of clusters 3 and 4 (N1 group) or cells of cluster 1 (N2 group).

Using unsupervised hierarchical clustering on the expression profiles, we identified several clusters of cells within the pre- and post-capture data. To classify these cell clusters accordingly to cell types, we further compared the cluster DEGs with the cell-type markers known from the reference data sets. As the reference data sets, we used three sets of RNA-seq data containing purified cortical neurons, astrocytes, microglia, endothelial cells, and oligodendrocytes (van de Leemput et al. 2014; Darmanis et al. 2015; Song et al. 2017). The cell type markers in the Darmanis et al. (2015) and Song et al. (2017) data sets were determined by Seurat as being differentially expressed between the cell types in each data set (FDR ≤ 0.05). We also extended the set of neuronal markers by adding the markers from van de Leemput et al. (2014) (reported with FDR ≤ 0.05), after the removal of genes highly expressed in other cell types, based on Zhang et al. (2014) expression data (using a twofold cutoff). We combined all targeted TF markers and used only unique markers for each cell type. There were in total 131 targeted TF markers present in the reference data sets, with 53 of them being present among 155 post-capture DEGs (Supplemental Fig. S2B,E) and 34 of the marker TFs being present among 129 pre-capture TF DEGs (Supplemental Fig. S3D). The significance of the identified TF markers in pre- and post-capture data sets was assessed by comparing the sets of TF markers in each data set to the sets of the referenced TF markers using a hypergeometric test (Supplemental Table S3).

### Gene regulatory network analysis

The coexpression GRNs were constructed with the bigScale package using default parameters by retaining only significant correlations with absolute value of Pearson coefficient > 0.8 (Iacono et al. 2019). The pre-capture, whole-transcriptome GRN consisted of 4286 nodes (genes) and 18,851 edges/connections between coexpressed genes. We also built the post-capture imputed GRN by extending the pre-capture libraries by the expression of 731 capture TFs rescaled for the differences in the sizes of pre- and post-capture libraries. The imputed GRN had 6365 nodes and 39,101 edges. For the plotting, we used only the cluster-specific GRNs, including DEGs that are up-regulated in ≥ 50% of the cluster cells (FDR ≤ 0.05). These were defined as genes differentially expressed among the pre-capture or imputed GRN genes either against all other clusters or against another single cluster. In case of differential expression in several comparisons between different clusters, DEGs were assigned to the cluster with the highest fold change. We defined cluster-specific TFs as those that were differentially expressed among the capture TFs in ≥ 50% of cluster cells. We retained only subnetworks with three or more nodes. To assess the significance of GRN enrichment in capture TFs, we used the network enrichment analysis (NEA) (Alexeyenko et al. 2012), which estimates the level of connectivity of a gene set (TFs) to the rest of the GRN by generating permuted networks with preserved degree distribution.

### Further GRN analysis (TF PPI and TF targets)

To explore the enrichment of the cluster subnetworks with TF PPIs, we used the “Transcription factor PPI” database of the Enrichr web server (Kuleshov et al. 2016). For this analysis, the cluster subnetworks were defined as those including the cluster-associated genes on the plotted GRNs: DEGs and captured TFs with ≥ 20% of their neighbors being the cluster DEGs. We plotted the top TF PPI terms of targeted TFs, having false discovery rate (FDR) ≤ 0.05 for the imputed GRN (and respective terms in pre-capture GRN). The significance of the difference between the number of genes in the reported pre- and post-capture TF PPIs was assessed with exact Wilcoxon-signed-rank test. The lists of potential TF targets were combined from two sources. First, we collected TFs and their targets from neuron and astrocyte networks (https://github.com/marbach/genecircuits) (Marbach et al. 2016), in which the tissue-specific gene regulatory networks had been inferred by combining transcription factor sequence motifs with activity data for promoters and enhancers from the FANTOM5 project. To reduce the number of potentially false positive targets, we used the interactions with high evidence scores with edge weights ≥ 0.1 (Misselbeck et al. 2019). Second, we predicted TF-target links specific to early stages in neuronal maturation from postmortem human brains (Colantuoni et al. 2011). We downloaded processed BrainCloud gene expression data from the NCBI Gene Expression Omnibus (GEO; https://www.ncbi.nlm.nih.gov/geo/) (GSE30272) and selected 70 samples from fetal to early postnatal stages (<1 yr old). On these we built coexpression networks with TFs as hub nodes by using ARACNE (Lachmann et al. 2016) with default parameters and selected significant TF-target links at *P*-value < 10^−7^. The significance of the enrichment of post-capture GRN in TF targets was assessed with the Fisher's exact test using the 2 × 2 contingency table.

### Pseudotime analysis

The pseudotime trajectories were estimated with the *destiny* package (Angerer et al. 2016) on log_2_-transformed counts after adding one count and scaling for the size factor for each library. Cells were ordered by pseudotime using the Slingshot package, specifying the initial cluster through inspection of TFs differentially expressed in each cluster with relation to known maturation stages (Street et al. 2018).

### Immunocytochemistry

iPSC-derived cultures were grown on coverslips and fixed in 4% paraformaldehyde/4% sucrose (w/v) in phosphate-buffered saline (PBS) solution. Cells were permeabilized in PBS with 0.4% v/v Triton X-100 for 2 × 7 min at room temperature (RT), then blocked in PBS with 10% v/v goat serum for 2 h at RT. GAD2 mouse monoclonal primary antibody (Chemicon, MAB351), diluted in PBS with 5% v/v goat serum (1:250), was applied for 2 h at RT. Goat anti-mouse Alexa Fluor 488 secondary antibody (Life Technologies A21131), diluted in PBS with 5% v/v goat serum (1:1000), was applied for 1 h at RT. Coverslips were washed three times for 5 min with PBS after each antibody application. Coverslips were then incubated with DAPI (Thermo Fisher Scientific; 1:5000 in PBS) for 5 min before being washed in PBS and briefly in dH_2_O, then mounted using Prolong Diamond Anti-Fade Mounting Solution (Thermo Fisher Scientific). Images were acquired on an Olympus BX40 Epifluorescence microscope using HCImage imaging software and processed using ImageJ.

### Patch clamp electrophysiology

To record spontaneous synaptic currents, iPSC-derived cultures grown on coverslips were bathed in a Tyrode's solution (140 mM NaCl, 5 mM KCl, 2 mM CaCl, 2 mM MgCl, 10 mM HEPES, 10 mM glucose; pH 7.36, osmolarity 290 mOsm and maintained at 30°C), visualized under an upright microscope (Olympus BX51WI), and targeted for whole-cell patch clamp recording with glass pipettes (tip resistance 5–10 MΩ) that had been pulled from standard wall borosilicate capillaries (OD 1.2 mm, ID 0.69 mm with filament; Warner Instruments). Pipettes were filled with a cesium gluconate solution (140 mM CsGlu, 6 mM NaCl, 1 mM EGTA, 10 mM HEPES, 4 mM MgATP, 0.4 mM Na_3_GTP). Signals were recorded at 10 kHz using a CVB-7 headstage and Multiclamp 700B amplifier controlled via Clampex (Molecular Devices), and subsequently analyzed using Clampfit (Molecular Devices). Each cell was voltage clamped at the reversal potential for glutamatergic currents (0 mV) and GABA A receptor–mediated spontaneous currents were recorded over a period of 4 min.

## Data access

All raw and processed sequencing data generated in this study have been submitted to the NCBI Gene Expression Omnibus (GEO; https://www.ncbi.nlm.nih.gov/geo/) under accession numbers GSE157835, GSE168590, and GSE168626 combined into the reference Series GSE168634.

## Supplementary Material

Supplemental Material
