# Supplementary material for: Targeted single-cell RNA sequencing of transcription factors enhances the identification of cell types and trajectories
Source: Genome Res. 2021 Jun;31(6):1069–81. doi: 10.1101/gr.273961.120 (PMC8168586; doi:10.1101/gr.273961.120)
Supplement: Supplemental Material [file supp_31_6_1069__DC1.html]

Targeted single-cell RNA sequencing of transcription factors enhances the identification of cell types and trajectories — Supplemental Material 

# Targeted single-cell RNA sequencing of transcription factors enhances the identification of cell types and trajectories

## Supplemental Material

- Supplementary\_Information.pdf
