# Supplementary material for: Targeted single-cell RNA sequencing of transcription factors enhances the identification of cell types and trajectories
Source: Genome Res. 2021 Jun;31(6):1069–81. doi: 10.1101/gr.273961.120 (PMC8168586; doi:10.1101/gr.273961.120)
Supplement: Supplemental Material [file supp_gr.273961.120_Supplementary_Information.pdf]

Supplementary Information

Pokhilko A., et al. Targeted single-cell RNA sequencing of transcription factors enhances the identification of cell types and trajectories

Table of content

Supplementary Figures p. 2

Figure S1 p. 2

Figure S2 p. 3

Figure S3 p. 5

Figure S4 p. 7

Figure S5 p. 8

Figure S6 p. 15

Figure S7 p. 16

Figure S8 p. 17

Figure S9 p. 19

Figure S10 p. 21

Figure S11 p. 22

Supplementary Tables p. 23

Table S1 p. 23

Table S2 p. 39

Table S3 p. 43

Table S4 p. 44

References p. 44

Figure S1. Capture-Seq improves the quality of scRNA-seq libraries and enriches for targeted TFs. **a,** Post-capture enrichment of 585 captured TFs detected pre-capture. For each gene the enrichment was calculated a ratio between the average CPMs in the post- and pre-capture libraries, plotted in log2 scale after adding a pseudocount of 1(Curion et al. 2020). **b,** Linear correlation between the pre- and post-capture averaged expression (raw counts) of 585 common TFs. ERCC spike-ins targeted by scCapture-seq are shown in red.

a

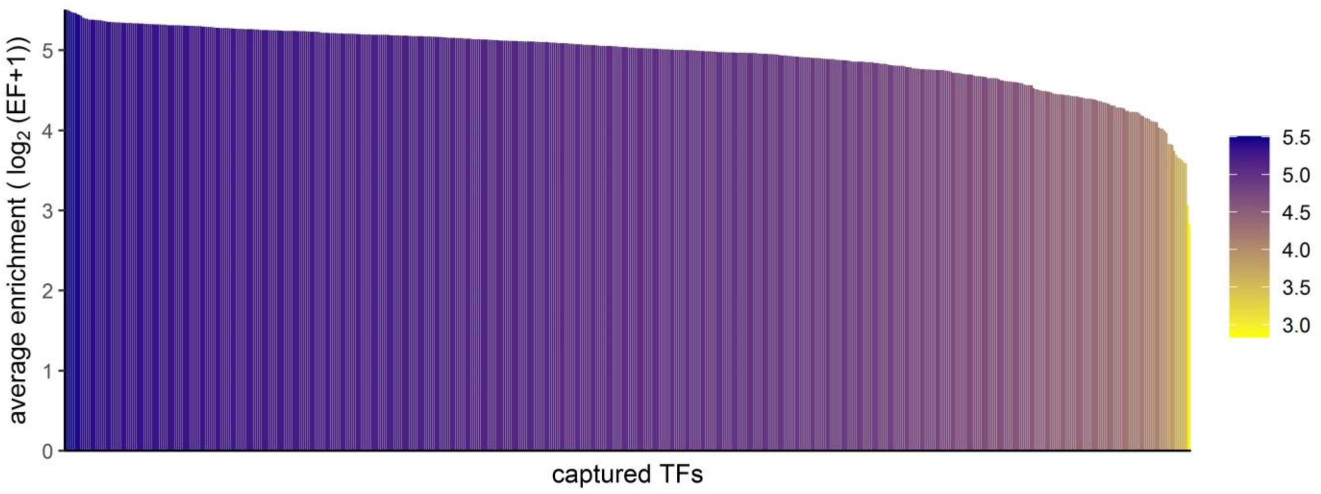

b

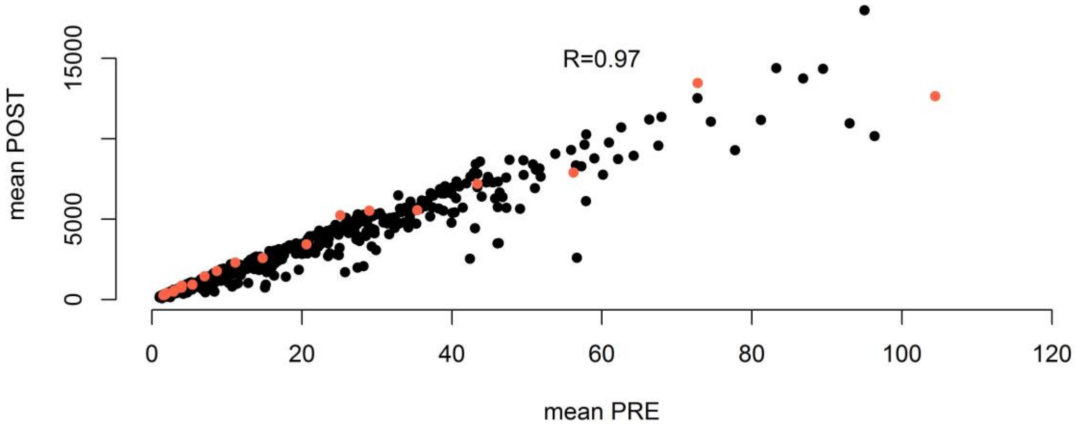

Figure S2. Characterization of cell clusters in pre- and post-capture libraries. **a,b**, Heat maps of cell type TF markers, which were differentially expressed post-capture (b), and their pre-capture expression (a). **c,d,** Heat maps of TF DEGs, which were differentially expressed post-capture (d,e) and their expression in pre-capture (c). Cells are ordered by post-capture clusters on a-d, as annotated above the heatmaps. The assignment of cells to pre-capture clusters is also annotated on a,c. The expression is log-normalized and centred. Only 52 out of 155 post-capture TF DEGs are labelled on c, d, for clarity of the heatmaps. The full list of TF DEGs is shown on e (the larger version of d).

a TF markers pre-capture

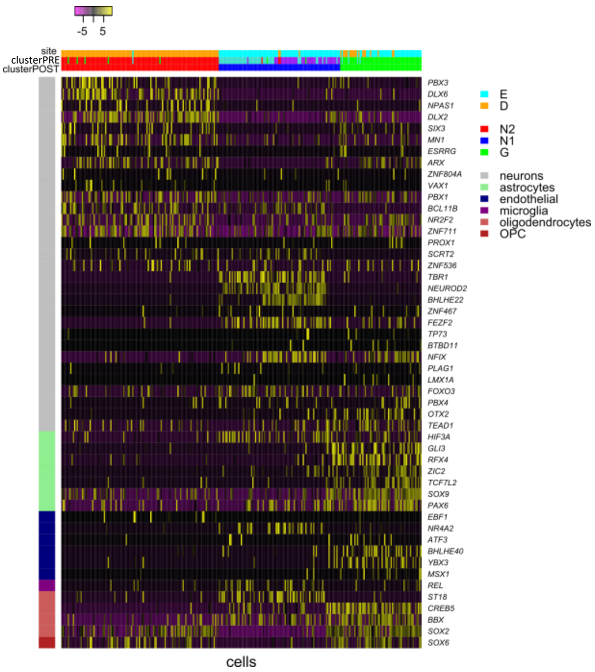

b TF markers post-capture

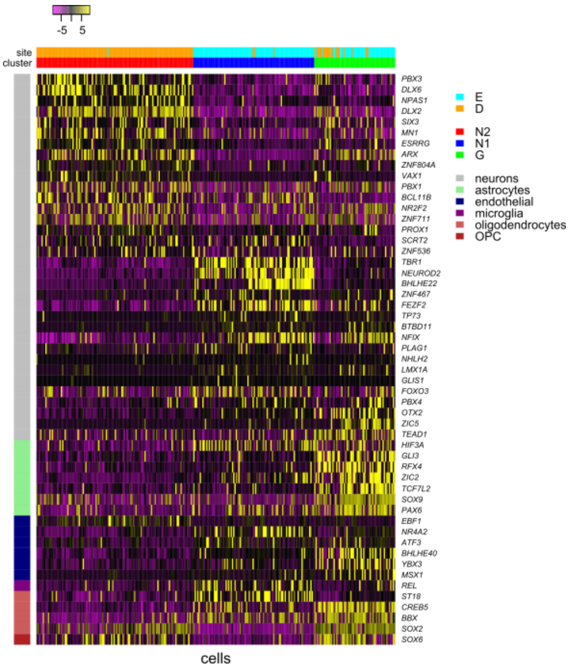

c pre-capture DEGs

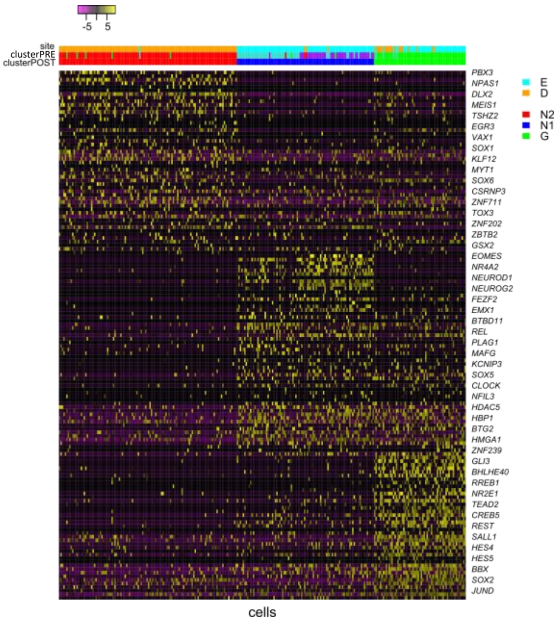

d post-capture DEGs

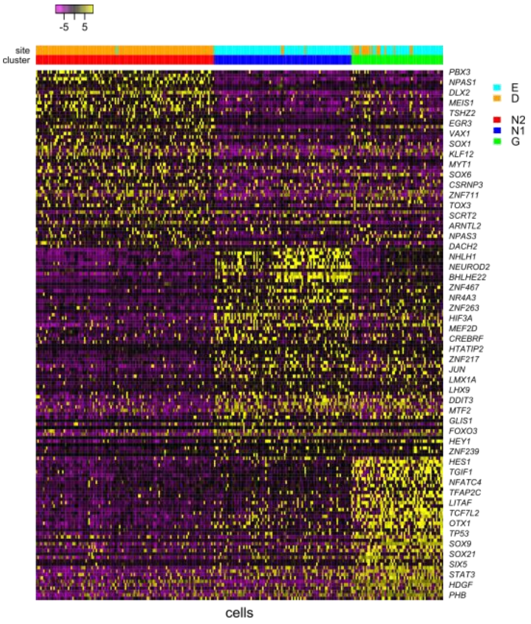

E

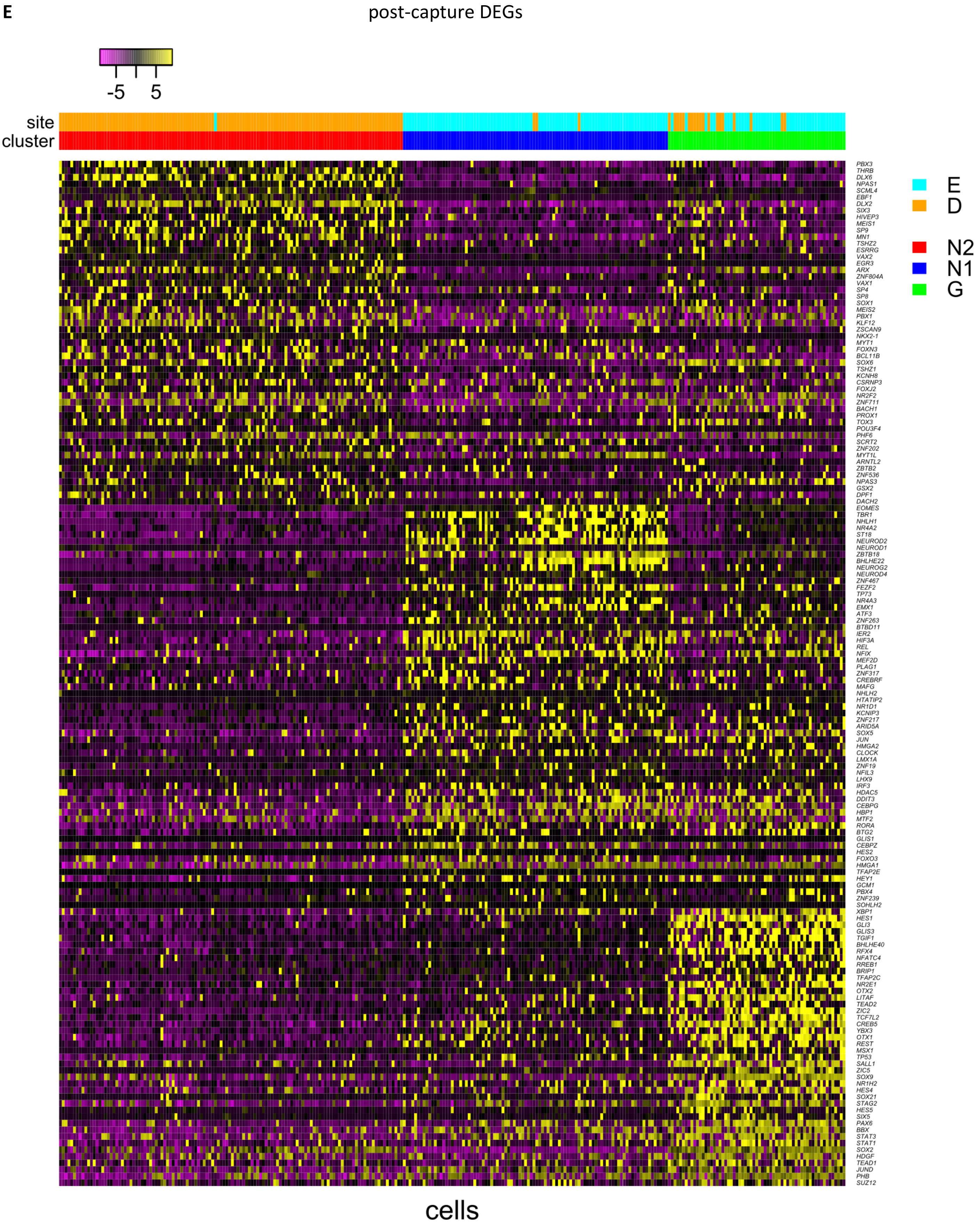
post-capture DEGs
-5
5
site
cluster
E
D
N2
N1
G
PBX3
THRB
DLX6
NPAS1
SCML4
EBF1
DLX2
SIX3
HIVEP3
MEIS1
SP9
MN1
TSHZ2
ESRRG
VAX2
EGR3
ARX
ZNF804A
VAX1
SP4
SP8
SOX1
MEIS2
PBX1
KLF12
ZSCAN9
NKX2-1
MYT1
FOXN3
BCL11B
SOX6
TSHZ1
KCNH8
CSRNP3
FOXJ2
NR2F2
ZNF711
BACH1
PROX1
TOX3
POU3F4
PHF6
SCRT2
ZNF202
MYT1L
ARNTL2
ZBTB2
ZNF536
NPAS3
GSX2
DPF1
DACH2
EOMES
TBR1
NHLH1
NR4A2
ST18
NEUROD2
NEUROD1
ZBTB18
BHLHE22
NEUROG2
NEUROD4
ZNF467
FEZF2
TP73
NR4A3
EMX1
ATF3
ZNF263
BTBD11
IER2
HIF3A
REL
NFIX
MEF2D
PLAG1
ZNF317
CREBRF
MAFG
NHLH2
HTATIP2
NR1D1
KCNIP3
ZNF217
ARID5A
SOX5
JUN
HMGA2
CLOCK
LMX1A
ZNF19
NFIL3
LHX9
IRF3
HDAC5
DDIT3
CEBPG
HBP1
MTF2
RORA
BTG2
GLIS1
CEBPZ
HES2
FOXO3
HMGA1
TFAP2E
HEY1
GCM1
PBX4
ZNF239
SOHLH2
XBP1
HES1
GLI3
GLIS3
TGIF1
BHLHE40
RFX4
NFATC4
RREB1
BRIP1
TFAP2C
NR2E1
OTX2
LITAF
TEAD2
ZIC2
TCF7L2
CREB5
YBX3
OTX1
REST
MSX1
TP53
SALL1
ZIC5
SOX9
NR1H2
HES4
SOX21
STAG2
HES5
SIX5
PAX6
BBX
STAT3
STAT1
SOX2
HDGF
TEAD1
JUND
PHB
SUZ12
cells

Figure S3. Comparison of the clustering of pre- and post-capture TFs. The clustering and differential expression were performed in Seurat using pre- and post-capture expression of the captured TFs. **a,b,c**, Umaps of cell clusters using 585 pre-capture (a) or 731 post-capture TFs (b). **c** Clustering of 585 pre-capture TFs coloured by the post-capture clusters of the panel b, illustrating that cell identities are preserved between a and b. **d,e,** Heat maps of differentially expressed TFs for a and b. The capture improves the detection of TFs, resulting in a substantial enrichment with N2 DEGs (e). Only 43 out of 129 pre-capture TF DEGs are labelled on d, and 52 out of 155 post-capture TF DEGs are labelled on e, for clarity of the heatmaps. The full list of post-capture TF DEGs is shown on Fig. S2e (larger version of Fig. S3e), while the full list of pre-capture TF DEGs is shown on Fig. S3f (larger version of Fig. S3d).

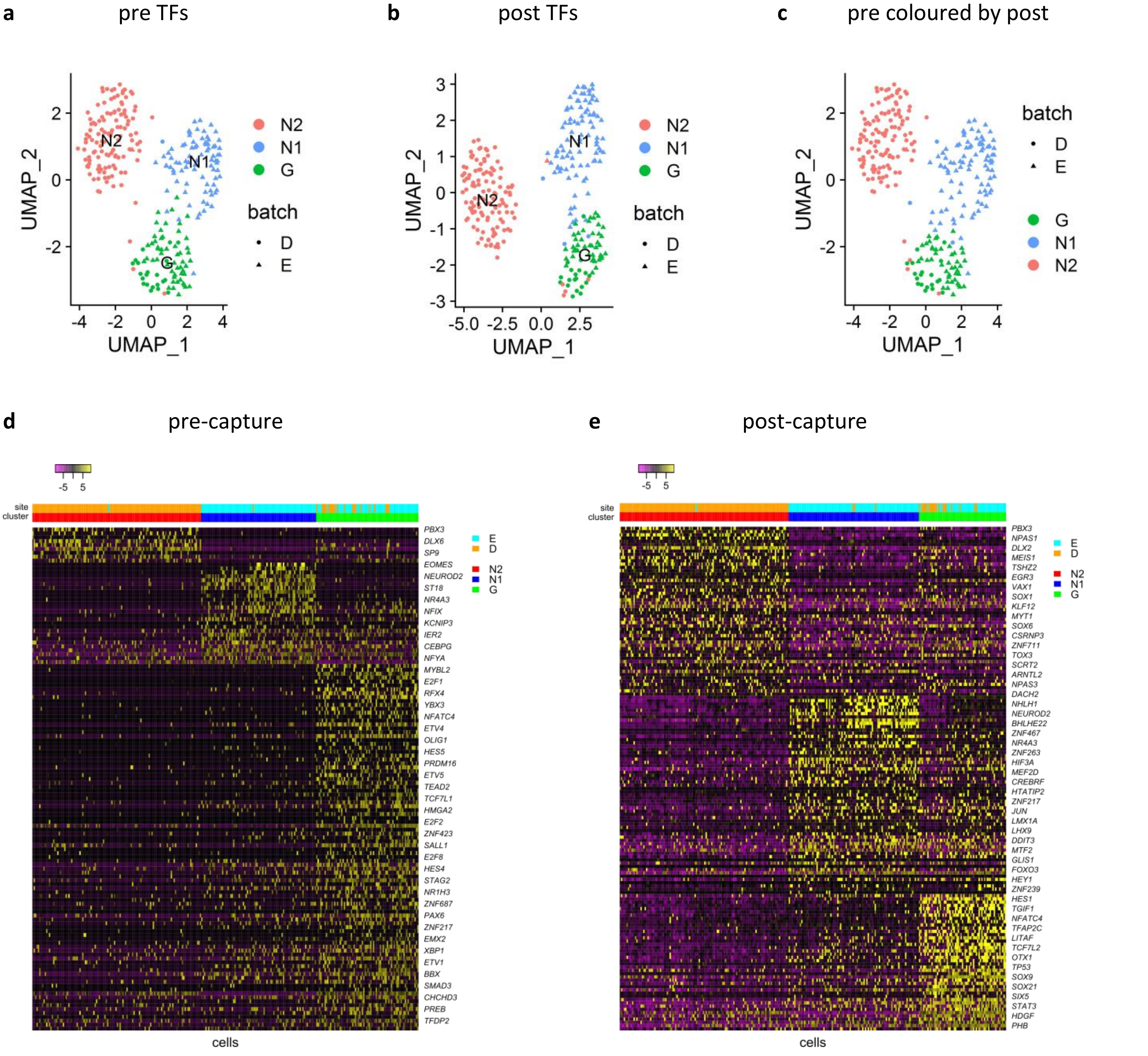

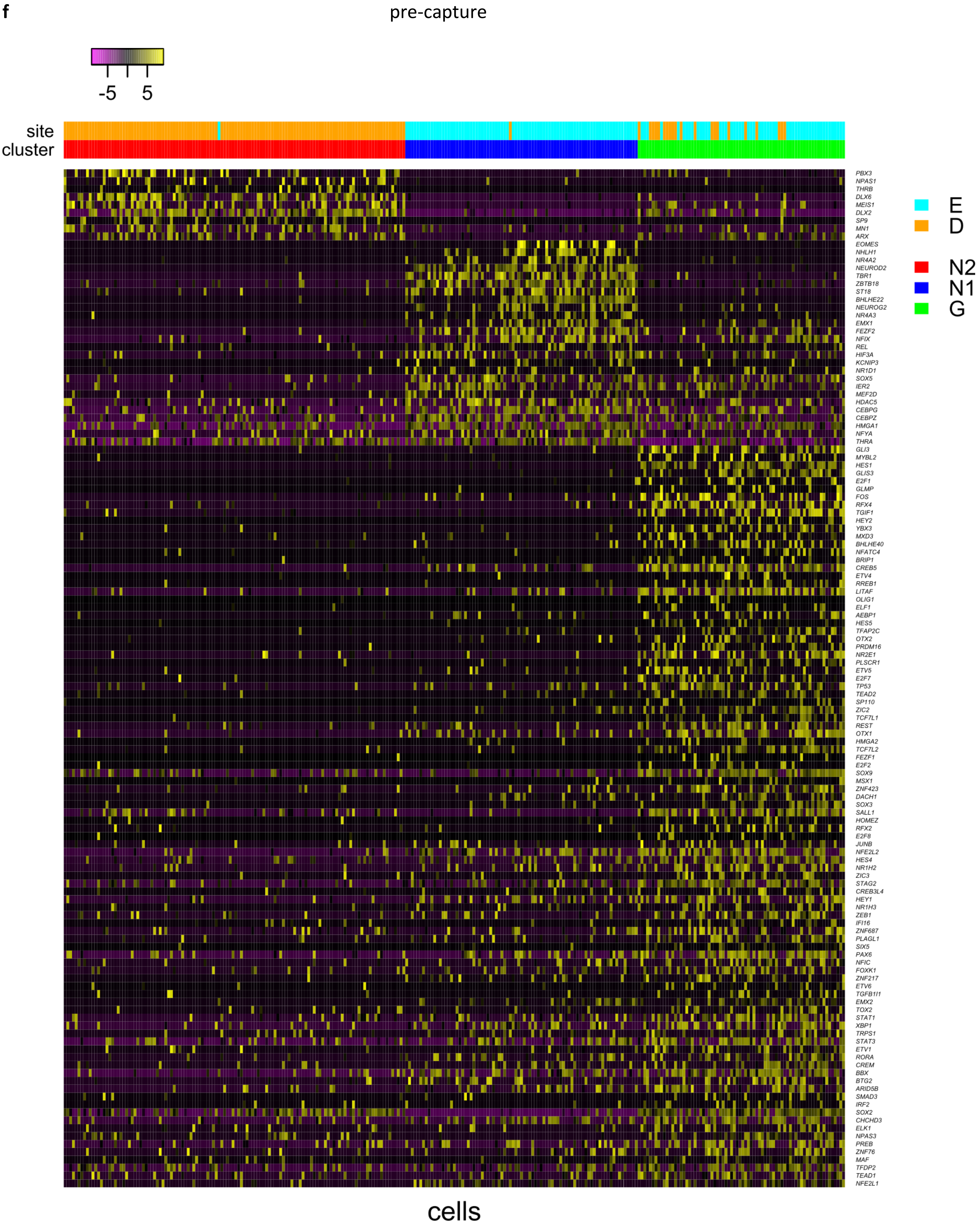
f
pre-capture
-5
5
site
cluster
E
D
N2
N1
G
PBX3
NPAS1
THRB
DLX6
MEIS1
DLX2
SP9
MN1
ARX
EOMES
NHLH1
NR4A2
NEUROD2
TBR1
ZBTB18
ST18
BHLHE22
NEUROG2
NR4A3
EMX1
FEZF2
NFIX
REL
HIF3A
KCNIP3
NR1D1
SOX5
IER2
MEF2D
HDAC5
CEBPG
CEBPZ
HMGA1
NFYA
THRA
GLI3
MYBL2
HES1
GLIS3
E2F1
GLMP
FOS
RFX4
TGIF1
HEY2
YBX3
MXD3
BHLHE40
NFATC4
BRIP1
CREB5
ETV4
RREB1
LITAF
OLIG1
ELF1
AEBP1
HES5
TFAP2C
OTX2
PRDM16
NR2E1
PLSCR1
ETV5
E2F7
TP53
TEAD2
SP110
ZIC2
TCF7L1
REST
OTX1
HMGA2
TCF7L2
FEZF1
E2F2
SOX9
MSX1
ZNF423
DACH1
SOX3
SALL1
HOMEZ
RFX2
E2F8
JUNB
NFE2L2
HES4
NR1H2
ZIC3
STAG2
CREB3L4
HEY1
NR1H3
ZEB1
IFI16
ZNF687
PLAGL1
SIX5
PAX6
NFIC
FOXK1
ZNF217
ETV6
TGFB1I1
EMX2
TOX2
STAT1
XBP1
TRPS1
STAT3
ETV1
RORA
CREM
BBX
BTG2
ARID5B
SMAD3
IRF2
SOX2
CHCHD3
ELK1
NPAS3
PREB
ZNF76
MAF
TFDP2
TEAD1
NFE2L1
cells

Figure S4. Numbers of cells expressing key TFs of the early differentiation stages from (Inoue et al. 2019) in each of 3 groups of cells (N1, N2 and G). The lab D N2 neurons, G cells and lab E N1 neurons were defined by clusters 1, 2 and 3+4 for pre-capture and by clusters N2, G and N1 for post-capture cells. Pre- and post-capture numbers of cells are shown by empty and solid circles, respectively.

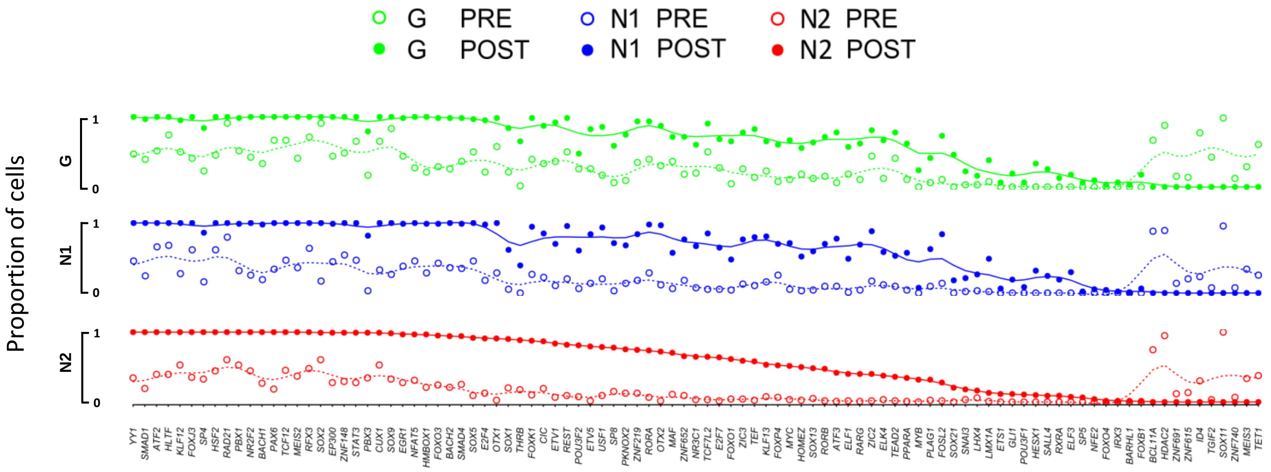

Figure S5. Expression of pre- and post-capture TFs in pseudotime space. **a** and **b** indicate TFs differentially expressed between clusters (a) or labs (b). a includes TF with clear maturation kinetics, such as *GFAP*, *HES1*, *HES5*, *PAX6*, *NR2E1*, *TCF7L2*, *OTX1*, corresponding to a radial glial proliferative stage and TFs of early stage of neuronal maturation (*NEUROD2*, *TBR1*, *BCL11B*, *BHLHE22*, *NFIX*). b shows some of TFs, which were differentially expressed between labs D and E and potentially involved in the developmental switch between N1 and N2. The expression was calculated as log2 of counts after adding 1.

a

Pre-capture

Post-capture

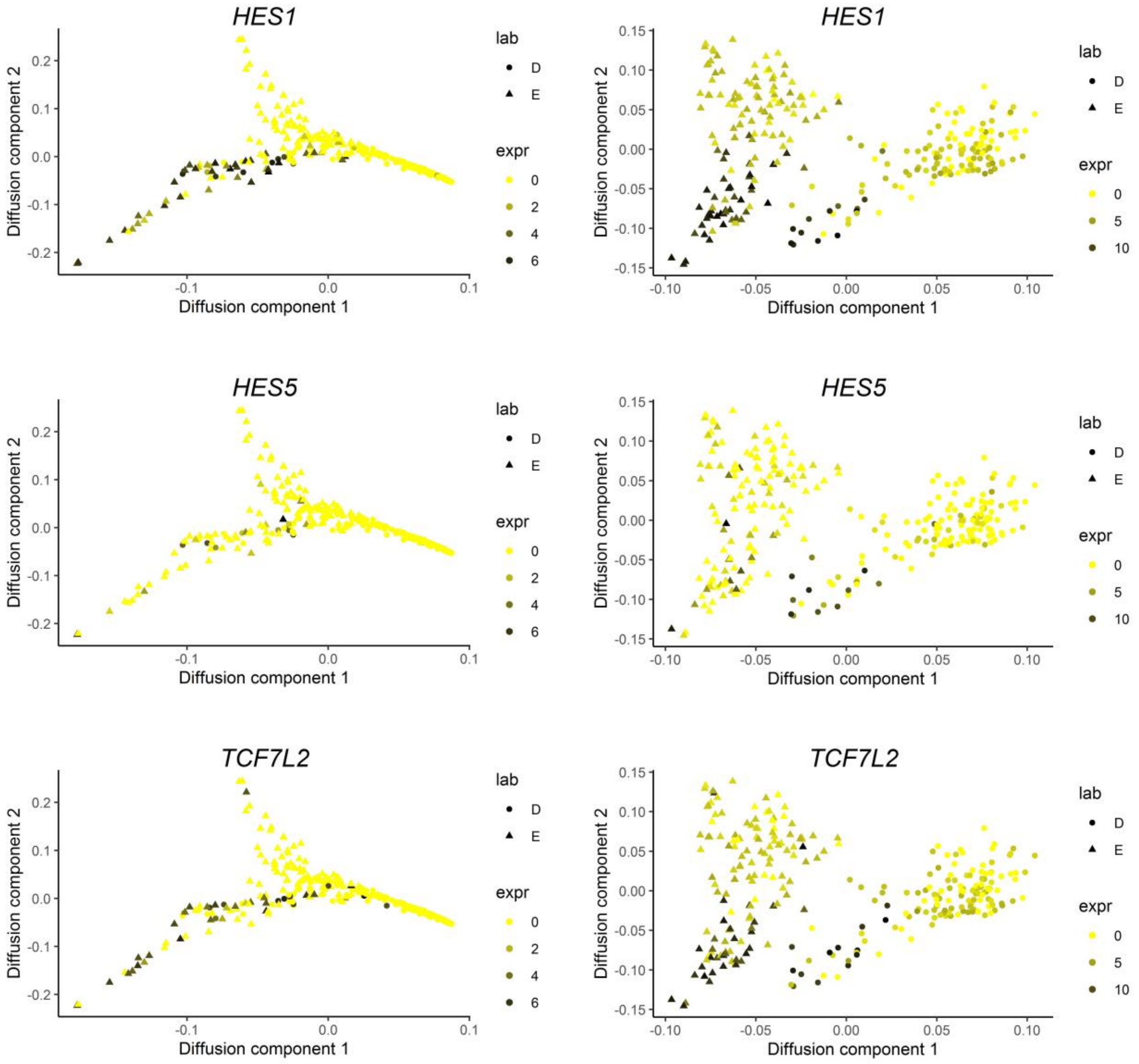

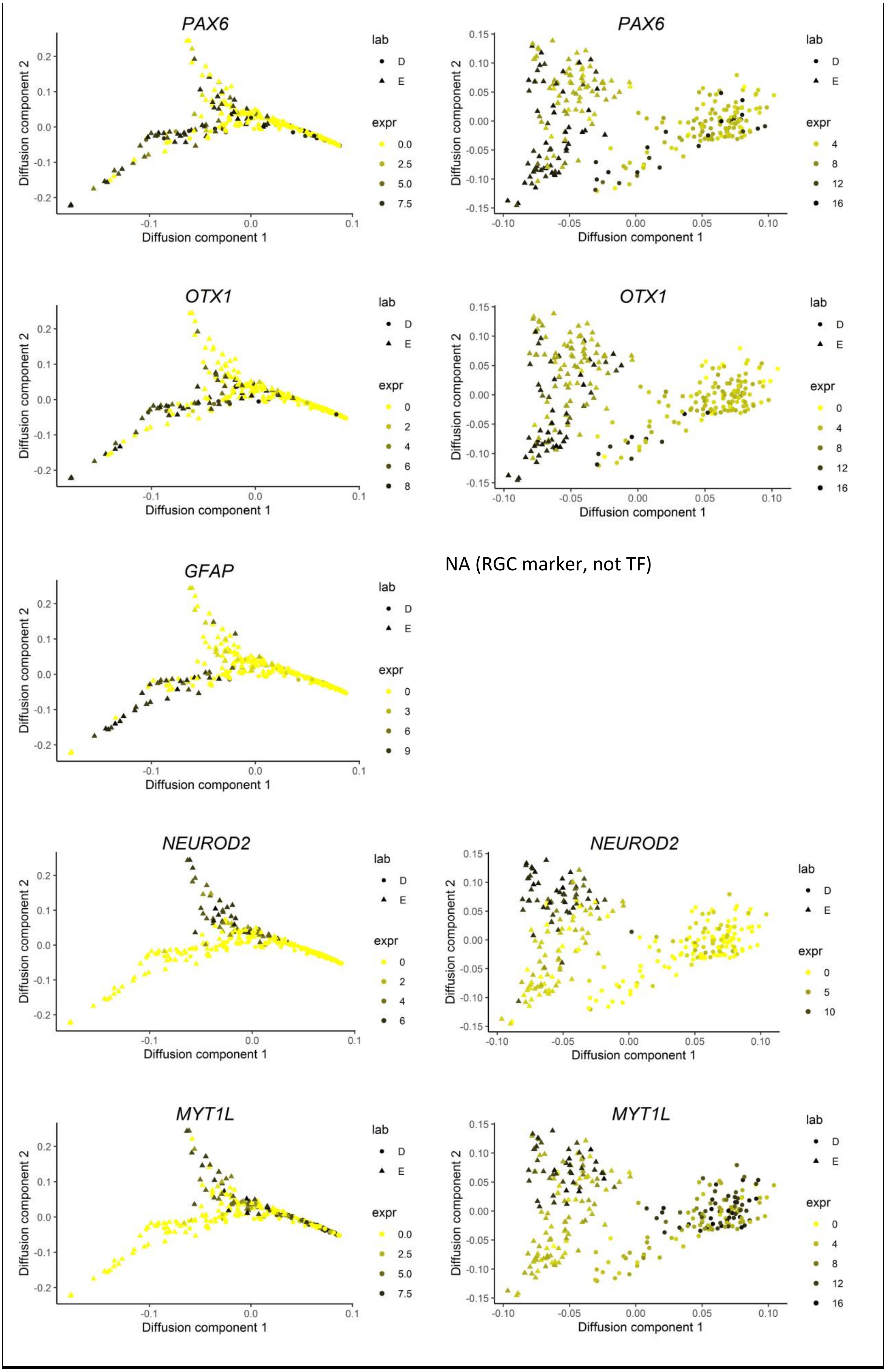
PAX6
Diffusion component 1
Diffusion component 2
lab
D
E
expr
0.0
2.5
5.0
7.5
PAX6
expr
4
8
12
16
OTX1
expr
0
2
4
6
8
OTX1
expr
0
4
8
12
16
GFAP
expr
0
3
6
9
NA (RGC marker, not TF)
NEUROD2
expr
0
2
4
6
NEUROD2
expr
0
5
10
MYT1L
expr
0.0
2.5
5.0
7.5
MYT1L
expr
0
4
8
12
16

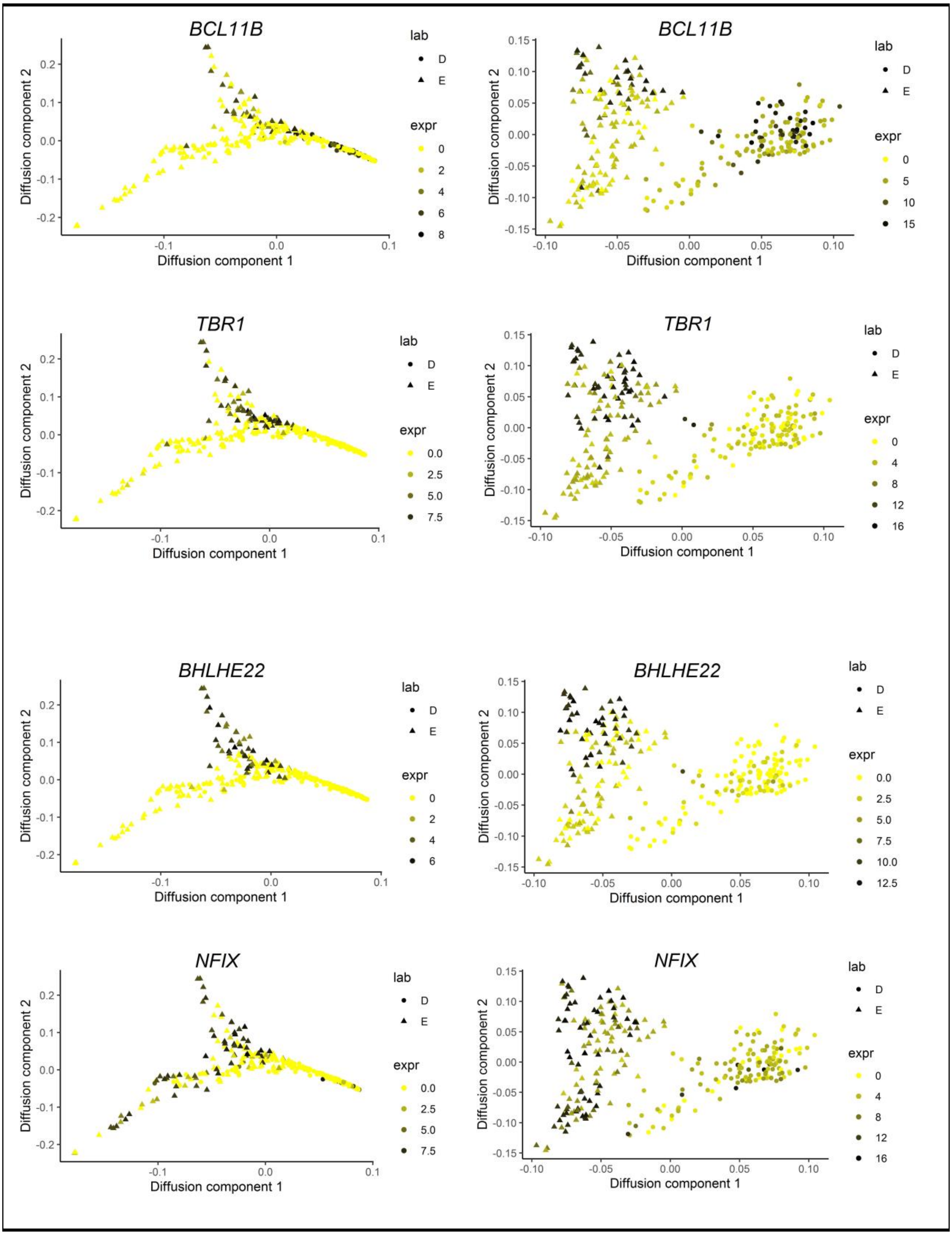
BCL11B
Diffusion component 1
Diffusion component 2
lab
D
E
expr
0
2
4
6
8
BCL11B
Diffusion component 1
Diffusion component 2
lab
D
E
expr
0
5
10
15
TBR1
Diffusion component 1
Diffusion component 2
lab
D
E
expr
0.0
2.5
5.0
7.5
TBR1
Diffusion component 1
Diffusion component 2
lab
D
E
expr
0
4
8
12
16
BHLHE22
Diffusion component 1
Diffusion component 2
lab
D
E
expr
0
2
4
6
BHLHE22
Diffusion component 1
Diffusion component 2
lab
D
E
expr
0.0
2.5
5.0
7.5
10.0
12.5
NFIX
Diffusion component 1
Diffusion component 2
lab
D
E
expr
0.0
2.5
5.0
7.5
NFIX
Diffusion component 1
Diffusion component 2
lab
D
E
expr
0
4
8
12
16

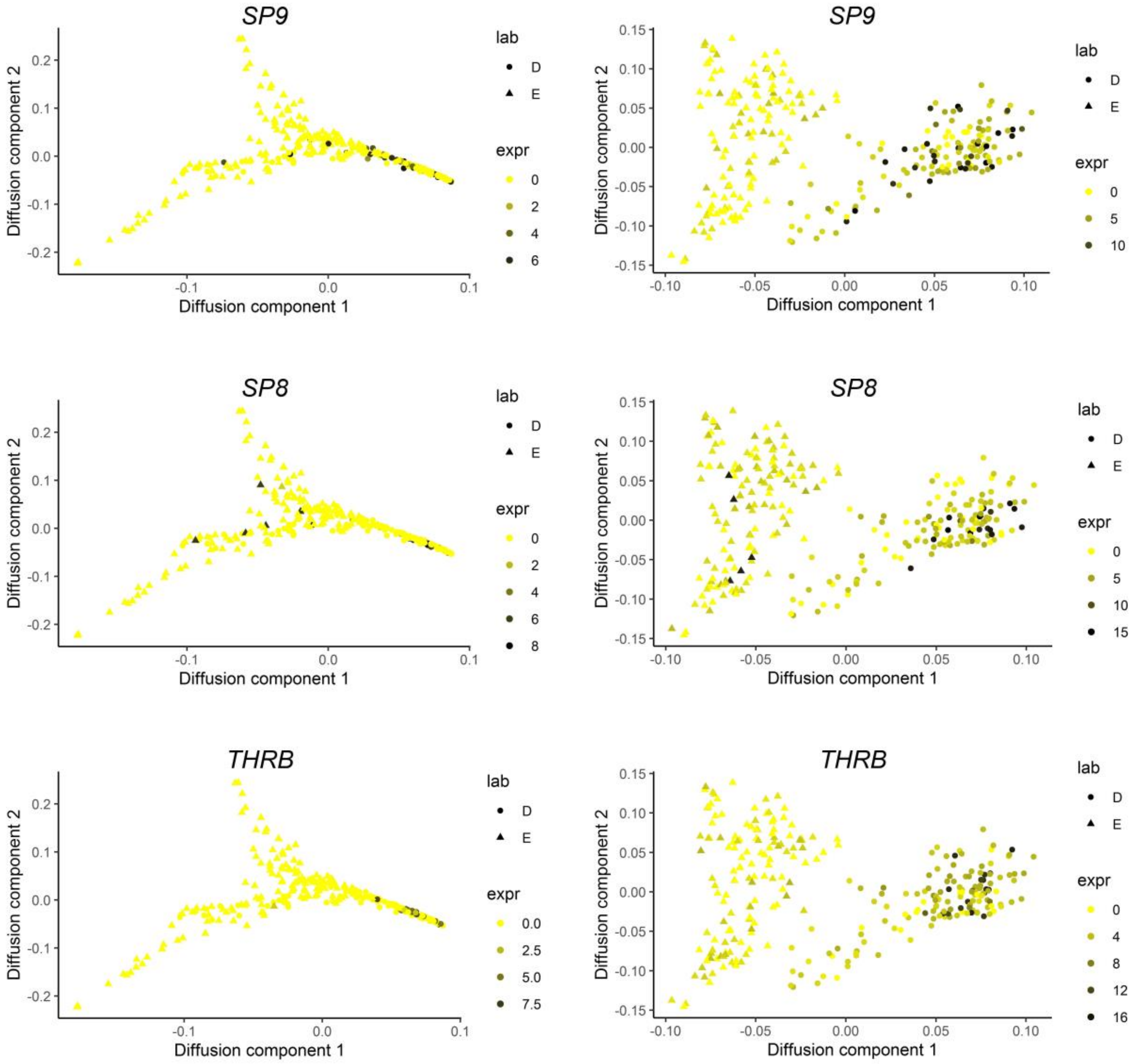
SP9
Diffusion component 1
Diffusion component 2
lab
D
E
expr
0
2
4
6
SP9
Diffusion component 1
Diffusion component 2
lab
D
E
expr
0
5
10
SP8
Diffusion component 1
Diffusion component 2
lab
D
E
expr
0
2
4
6
8
SP8
Diffusion component 1
Diffusion component 2
lab
D
E
expr
0
5
10
15
THRB
Diffusion component 1
Diffusion component 2
lab
D
E
expr
0.0
2.5
5.0
7.5
THRB
Diffusion component 1
Diffusion component 2
lab
D
E
expr
0
4
8
12
16

b

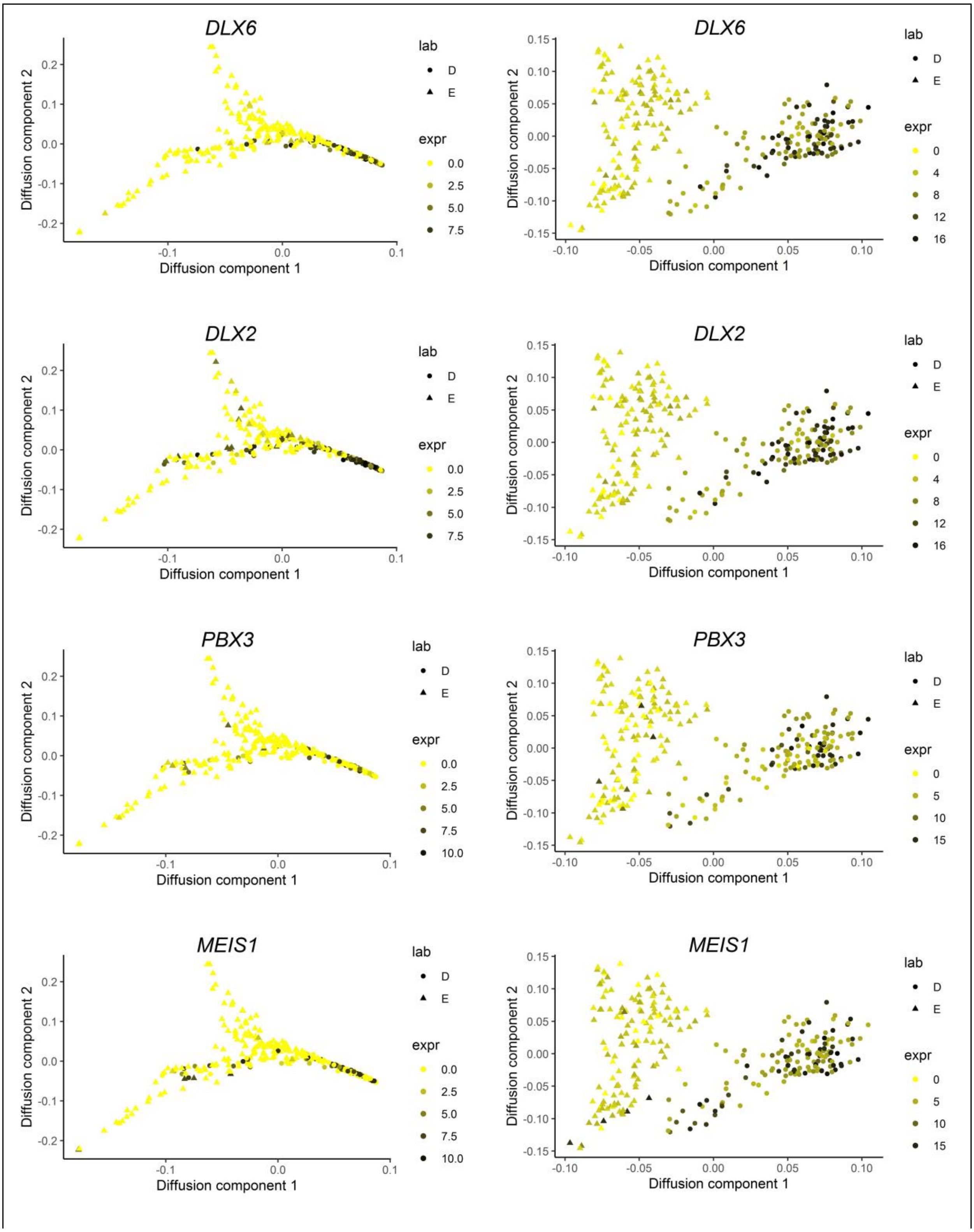
DLX6
Diffusion component 1
Diffusion component 2
lab
D
E
expr
0.0
2.5
5.0
7.5
DLX6
Diffusion component 1
Diffusion component 2
lab
D
E
expr
0
4
8
12
16
DLX2
Diffusion component 1
Diffusion component 2
lab
D
E
expr
0.0
2.5
5.0
7.5
DLX2
Diffusion component 1
Diffusion component 2
lab
D
E
expr
0
4
8
12
16
PBX3
Diffusion component 1
Diffusion component 2
lab
D
E
expr
0.0
2.5
5.0
7.5
10.0
PBX3
Diffusion component 1
Diffusion component 2
lab
D
E
expr
0
5
10
15
MEIS1
Diffusion component 1
Diffusion component 2
lab
D
E
expr
0.0
2.5
5.0
7.5
10.0
MEIS1
Diffusion component 1
Diffusion component 2
lab
D
E
expr
0
5
10
15

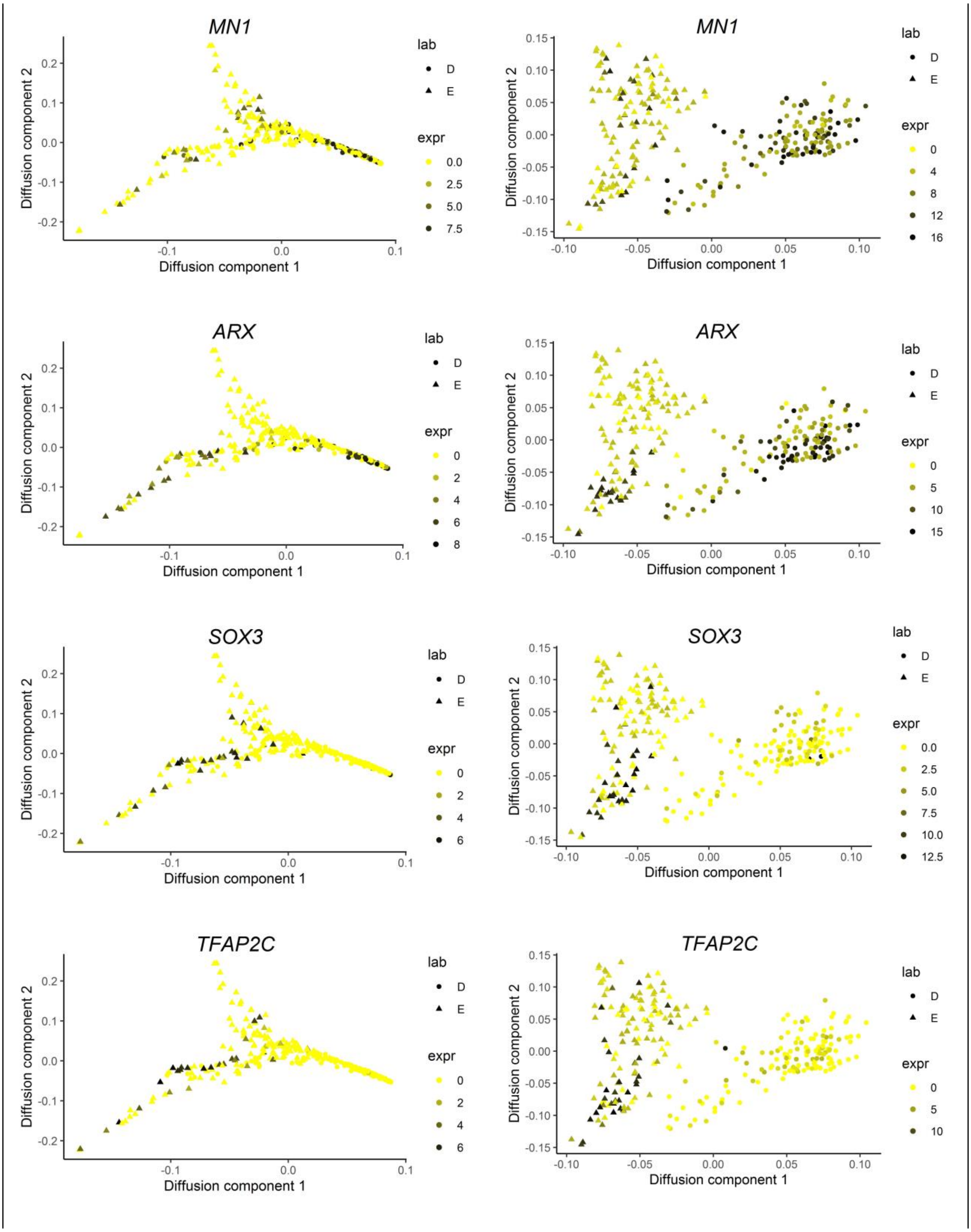
MN1
Diffusion component 1
Diffusion component 2
lab
D
E
expr
0.0
2.5
5.0
7.5
MN1
lab
D
E
expr
0
4
8
12
16
ARX
lab
D
E
expr
0
2
4
6
8
ARX
lab
D
E
expr
0
5
10
15
SOX3
lab
D
E
expr
0
2
4
6
SOX3
lab
D
E
expr
0.0
2.5
5.0
7.5
10.0
12.5
TFAP2C
lab
D
E
expr
0
2
4
6
TFAP2C
lab
D
E
expr
0
5
10

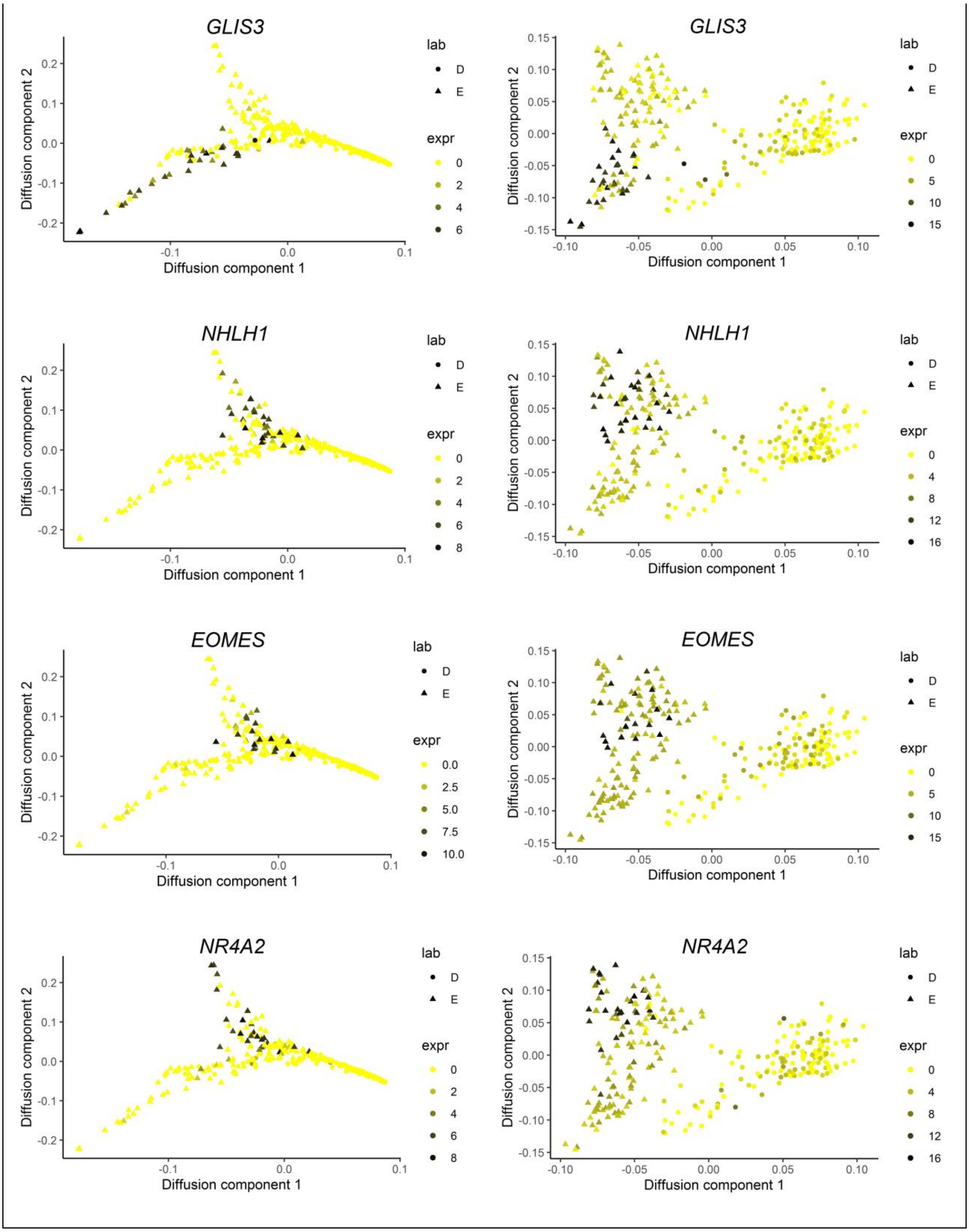
GLIS3
Diffusion component 2
Diffusion component 1
lab
D
E
expr
0
2
4
6
GLIS3
Diffusion component 2
Diffusion component 1
lab
D
E
expr
0
5
10
15
NHLH1
Diffusion component 2
Diffusion component 1
lab
D
E
expr
0
2
4
6
8
NHLH1
Diffusion component 2
Diffusion component 1
lab
D
E
expr
0
4
8
12
16
EOMES
Diffusion component 2
Diffusion component 1
lab
D
E
expr
0.0
2.5
5.0
7.5
10.0
EOMES
Diffusion component 2
Diffusion component 1
lab
D
E
expr
0
5
10
15
NR4A2
Diffusion component 2
Diffusion component 1
lab
D
E
expr
0
2
4
6
8
NR4A2
Diffusion component 2
Diffusion component 1
lab
D
E
expr
0
4
8
12
16

Figure S6. Volcano plots of TFs differentially expressed between Lab E and Lad D cells. **a**, Pre-capture TF DEGs between N1 and N2 neurons (based on expression of 585 targeted TFs). **b**, post-capture TF DEGs between G cells from lab D and lab E. Significant TF DEGs are labelled (FDR ≤ 0.05, natural log fold changes ≥ 0.5).

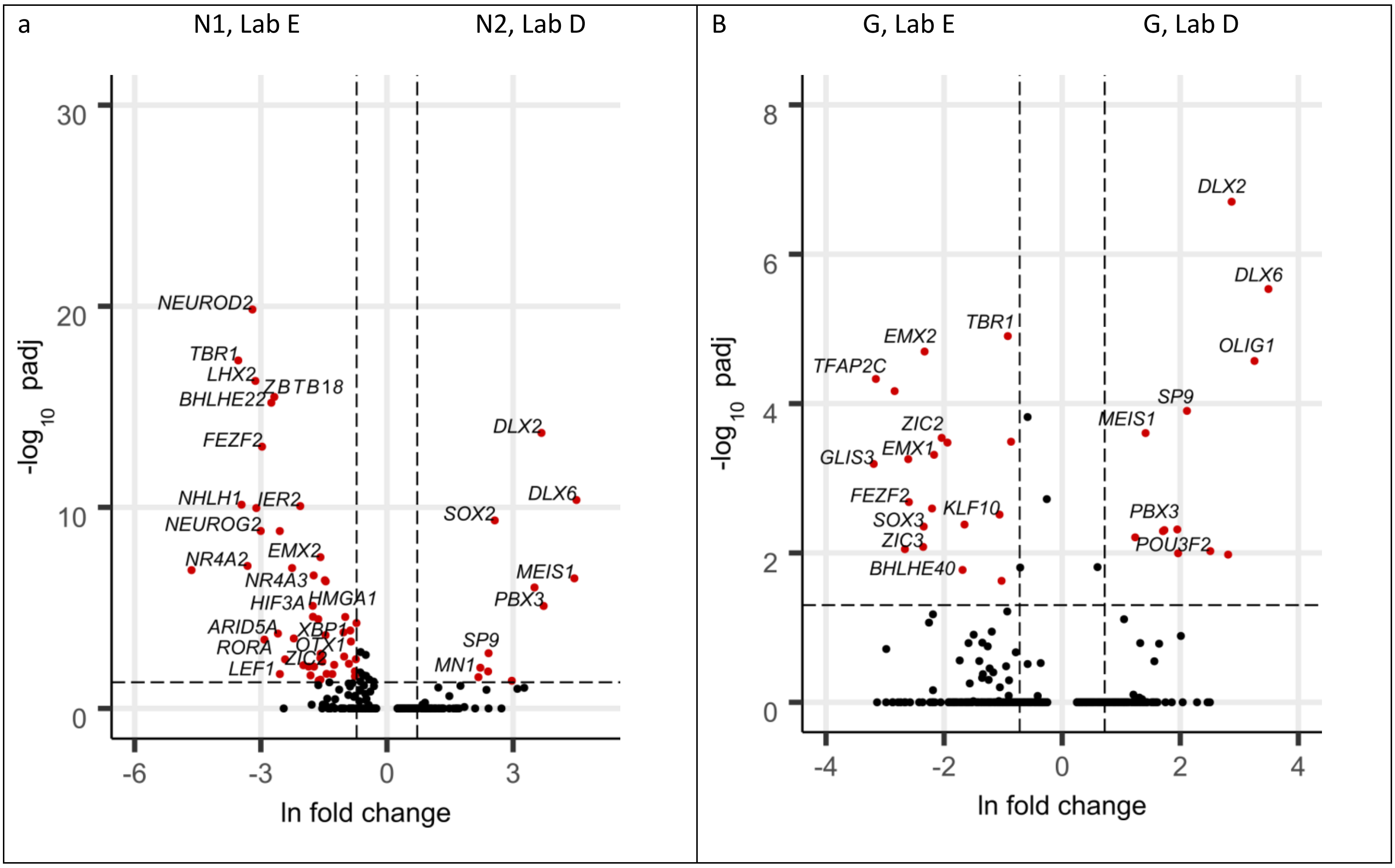

Figure S7. Analysis of GRN TFs related to retinoic acid (RA) signalling. **a**, Co-expression subnetwork of the GRN of Fig. 4b, including TF PPIs involved in retinoic acid signalling. The network was built using TFs belonging to *RARA*, *RXRA* and *CEBPB* terms of TF PPI of cluster N2 (Fig. 4e, Table S2). The TF PPIs are marked with orange borders and they are plotted together with the neighbours on the GRN of Fig. 4b. The network genes are coloured as indicated in the legend. Thicker edges show direct connections between the TF PPIs and GRN genes. Orange and blue colour of the edges correspond to positive and negative correlation, respectively. Noticeably, many of N2 DEGs (e.g., *DLX2*, *DLX6*) are negatively correlated to RA TFs (e.g., *RORA*, *NR1H2*, *ZNF423*). **b**, Heatmaps of post-capture log2 expression of the TFs from RA-related TF PPIs. The cells from different labs are ordered by pseudotime.

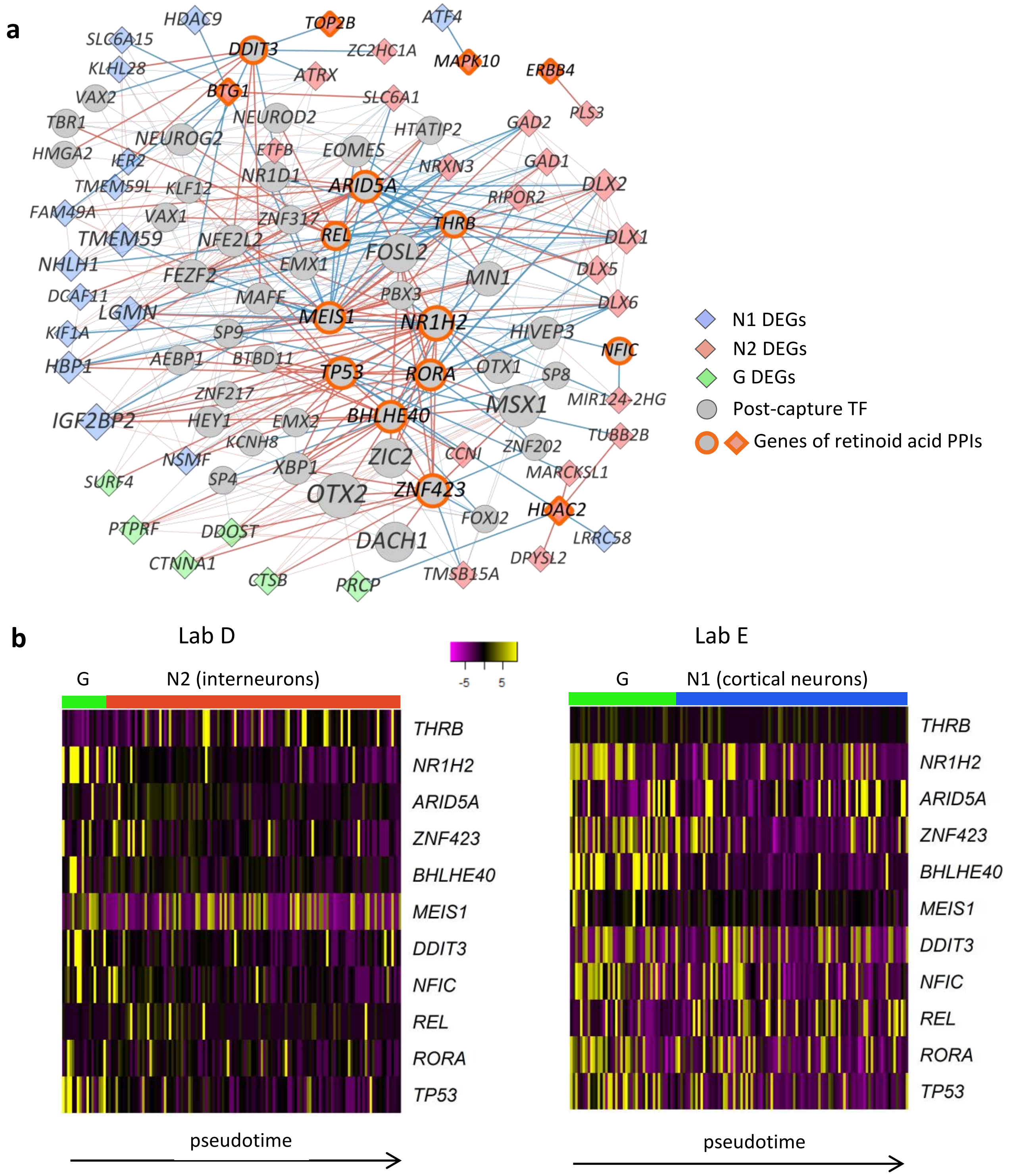

Figure S8. Validation of scCapture-seq with intestinal stromal cells from UC patients. Capture-Seq improves the quality of scRNA-seq libraries and enriches for targeted TFs. **a,** Linear correlation between the pre- and post-capture averaged expression (raw counts) of 530 common TFs, expressed both pre- and post-capture. ERCC spike-ins are shown in red. **b**, Post-capture enrichment of the captured TFs detected pre-capture. For each gene the enrichment was calculated a ratio between the average CPMs in the post- and pre-capture libraries, plotted in log2 scale after adding a pseudocount of 1 (Curion et al. 2020). **c,** Increase in the number of TFs detected per cell post-capture. **d**, UMAPs of pre - (left) and post- (middle) captured clusters show 2 cellular clusters. UMAPs were constructed using Seurat package (Stuart et al. 2019). The right panel shows post-capture cells coloured by pre-capture clusters. **e, f**. Feature plots of differentially expressed TFs pre- (e) and post- (f) capture in low-dimensional space, using Seurat package on ln-transformed expression values

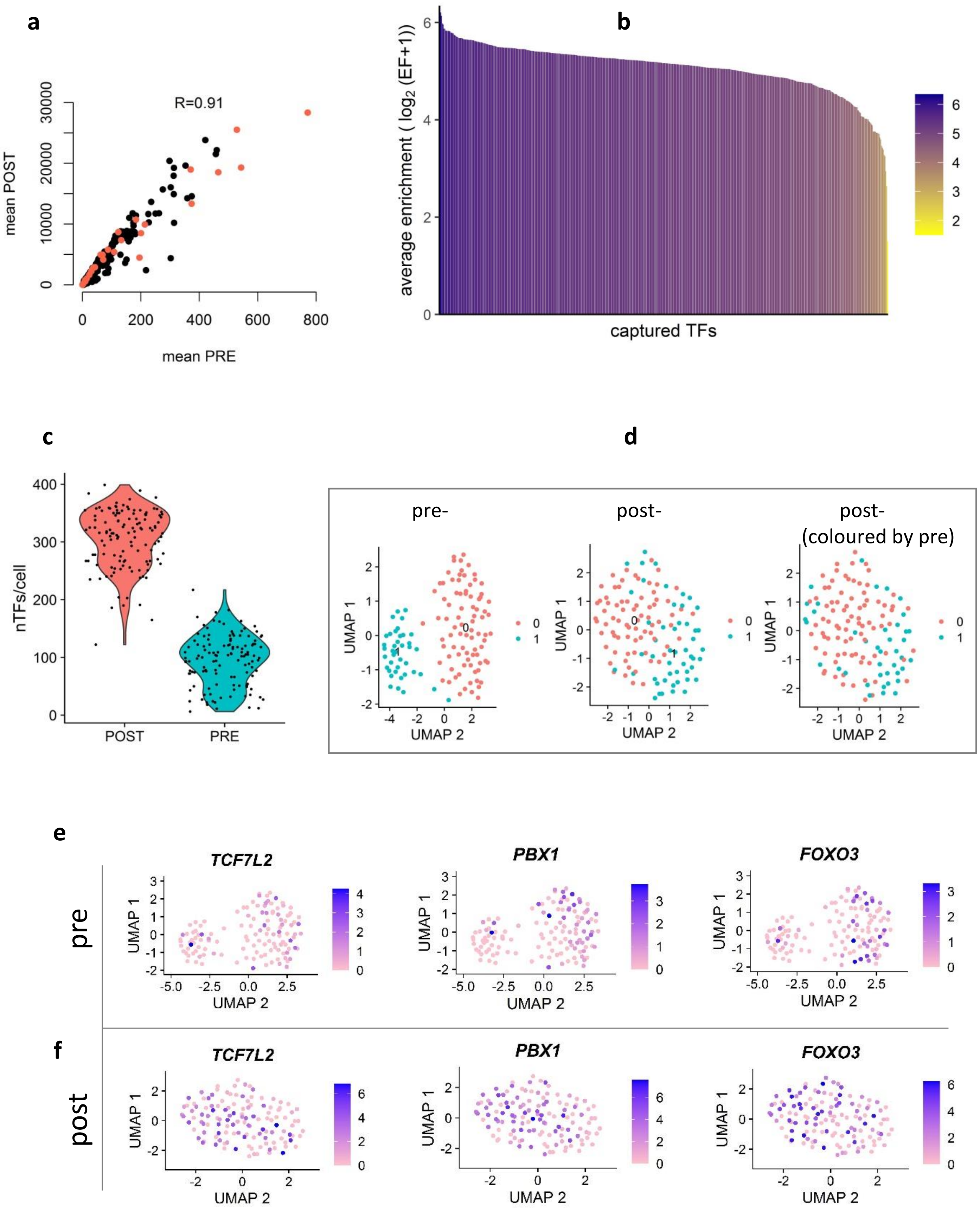
a
R=0.91
mean POST
mean PRE
0
10000
20000
30000
0
200
400
600
800
b
average enrichment ($\log_2$ (EF+1))
captured TFs
c
nTFs/cell
POST
PRE
d
pre-
post-
post-
(coloured by pre)
UMAP 1
UMAP 2
e
pre
TCF7L2
PBX1
FOXO3
f
post

Figure S9. Validation of scCapture-seq with NG capture in iPSC cortical neurons. **a,** Linear correlation between the pre- and post-capture averaged expression (raw counts) of 71 common NGs, expressed both pre- and post-capture. ERCC spike-ins are shown in red. **b**, Post-capture enrichment of the common NGs. **c,** Increase in the number of TFs detected per cell post-capture. **d**, UMAPs of pre - (left) and post- (middle) captured clusters, as well as post-capture cells coloured by pre-capture clusters (right). The clustering was performed on all (pre-capture) or targeted (post-capture) expressed transcripts. **e**, Heatmap of post-capture NG DEGs. **f**. Typical feature plots of NG DEGs.

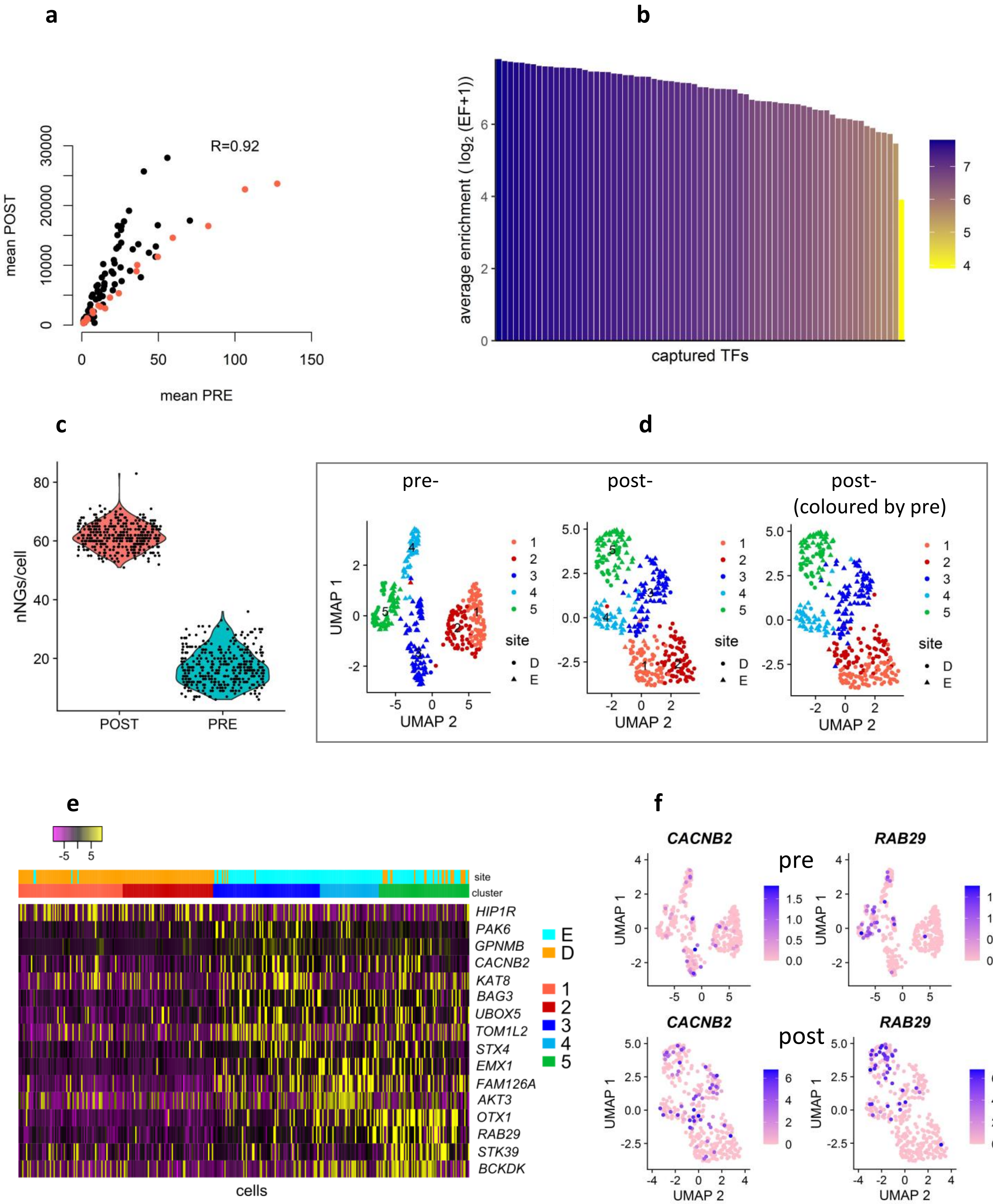
a
b
R=0.92
mean POST
mean PRE
average enrichment ($\log_2$ (EF+1))
captured TFs
c
d
nNGs/cell
POST
PRE
pre-
post-
post-
(coloured by pre)
UMAP 1
UMAP 2
site
D
E
e
f
site
cluster
HIP1R
PAK6
GPNMB
CACNB2
KAT8
BAG3
UBOX5
TOM1L2
STX4
EMX1
FAM126A
AKT3
OTX1
RAB29
STK39
BCKDK
cells
CACNB2
RAB29
pre
post

Figure S10. Characterization of cell clusters in pre-capture libraries of iPSC-derived cultures of cortical neurons used for NG capture. Heat map shows cell type marker DEGs. The expression is log-normalized and centred.

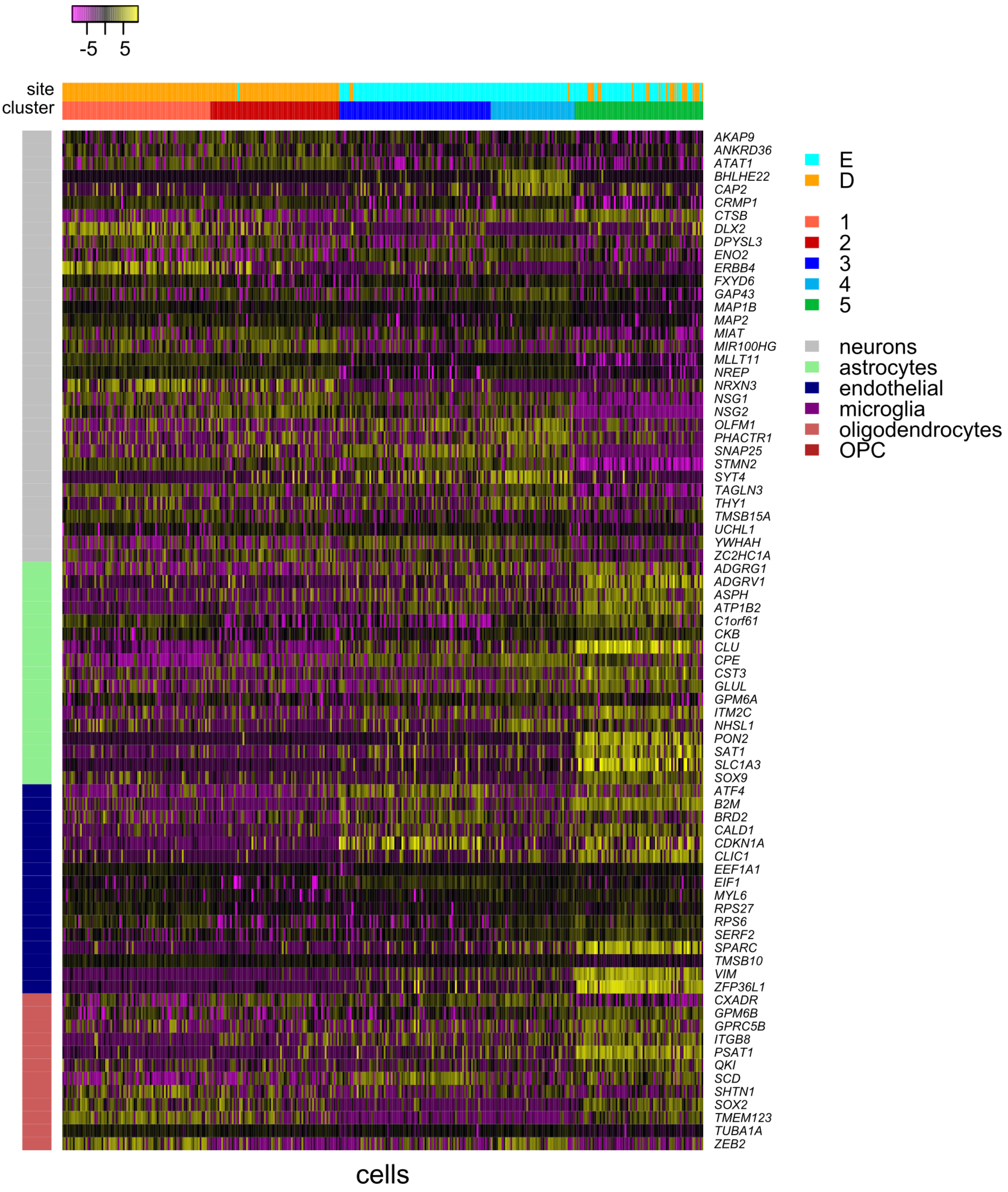

Figure S11. Comparison of read mapping between neuronal and gut libraries generated using SMART-seq2 or 10x Genomics sequencing. The mapping is shown for *SP1* gene expressed in both cell types. The figure was generated using UCSC genomic browser, for typical pre and post TF capture libraries of neuronal and gut cell, as indicated. There was comparable increase of read numbers post-capture, with more even coverage of 3' and 5' ends in the SMART-seq2 neuronal libraries.

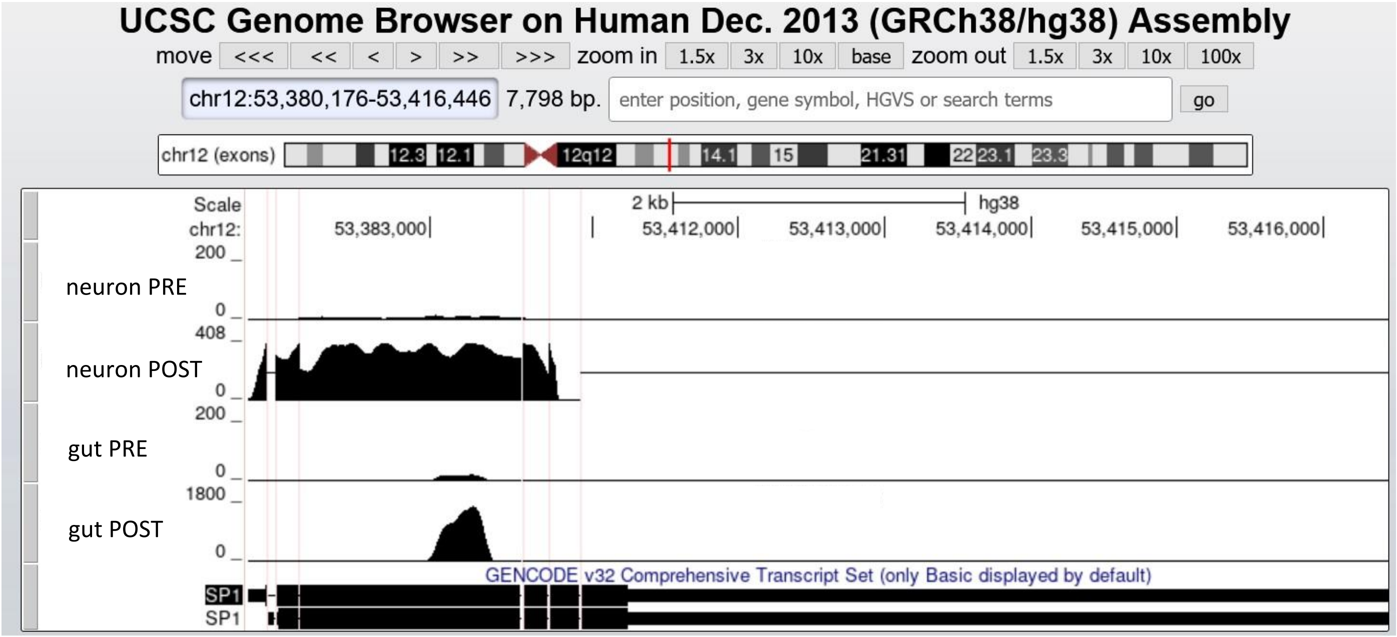

Table S1. GRN TFs with their targets. The targets are predicted based on neuron and astrocyte networks of (Marbach et al. 2016); or based on BrainSpan and BrainCloud datasets (Colantuoni et al. 2011; Lachmann et al. 2016). The table consists of 2 parts, marked by thick borders: first part corresponds to pre-capture GRN and second part corresponds to post-capture imputed GRN

TF	targets Marbach neurons pre-capture	targets Marbach astrocytes pre-capture	targets BrainSpan, BrainCloud
BBX	-	*CREB5, NRCAM*	*GNPDA1, SLC25A1*
BHLHE22	-	-	*KDM5B, CXADR, SLC6A15, IFITM2, MTSS1, GRHPR, LGALS3BP, GFAP, DPYSL3, FAM126A, CTSA, BCL11A, DLX2, DOK5*
CEBPG	*SALL1, NTRK2, GNG3, NRCAM, BCL11A*	*SALL1, NRCAM, NTRK2*	*GAD2*
CEBPZ	-	-	*GNG3, ARL6IP6, OTX1*
CREB5	*SOX2, PAX6, CTNNA1, NFIX, CDK6, DLX6, NKAIN4, BCAN, TMEM59L*	*PAX6, SOX2, DLX6, CTNNA1, CDK6, NFIX, CREB5*	*TP53I13, ARF1, CDK6, TMEM98*
DLX2	*FEZF2, KLHDC8A, SALL1, NRCAM, ELAVL4, LIX1, NTRK3, COL4A6*	*FEZF2, NRCAM, KLHDC8A, MAPK10, LIX1, SALL1, COL4A6*	*MGST1, ZNF704, RNASEH2A, BHLHE22, MCM4, SLC9A3R1, CXADR, SLC6A15, ATAT1, IFITM2, DUT, BTG1, GRN, ZNF608, LGALS3BP, GFAP, DPYSL3, AKAP9, SNCB, TOP2B, VPS52, TMEM35A, DLX1, NINJ1, SCRG1, TSPAN6, DOK5*
DLX6	*KLHDC8A, SALL1, NTRK3*	*KLHDC8A, SALL1*	-
FEZF2	-	-	*MGST1, RNASEH2A, SEMA4D, PCNA, STAT3*
HES1	-	-	*LTBP1, PLCE1, GMNN, MAPK10, GPC4, CNN3, TNC, OTX1, PDPN*

HES4	-	-	*PEPD, YIF1A, TUBA1B*
IER2	-	-	*PDE2A, ZWINT*
LHX2	*GAD2, TBR1, NRXN3, SYT4, LIX1, NHLH1, SALL1*	*TBR1, NRXN3, SEL1L3, LIX1, SYT4, SALL1*	*TP53I11, STOML2*
LITAF	-	-	*RNASEH2A, RAB6A, PGM2L1, LTBP1, TP53I13, ADGRV1, NOTCH2, NR2E1, GNG5, SALL1, PSME1, PTBP1*
MYT1L	-	-	*PHGDH, PAK5, SLC9A3R1, LRIG1, CXADR, TIMP1, SOX2, HSDL2, NPC2, GRHPR, MLC1, PDE1A, SLC39A1, NINJ1, NELL2, AIF1L, NKAIN4, CHL1, DOK5*
NEUROD2	*ZBTB18*	*ZBTB18*	*ATAT1, MTSS1, GNG3, SNAP25, BTG1, PPT1, MAPK10, NES, SNCB, BCL11A*
NFIX	-	-	*POLE3, OTX1*
NHLH1	*NES, DNAJB5, CTNNA2, BCAN, SOX2, SLC1A2, PPP2R5B, SLC9A3R1, STAT3, TCF4, TNC, ID3, PDPN*	*NES, DNAJB5, BCAN, SOX2, SEL1L3, SLC1A2, CTNNA2, TNC, LTBP1, STAT3, PDPN*	*LRRC55, GINS2, TYMS*
NR2E1	*SEMA6D, NEUROD2, TCF4, NR2E1, RCN1, FAM49A, SALL1, DLX1, NLGN1, SEL1L3, MLC1, PSAT1, DLX2*	*SEMA6D, NR2E1, NLGN1, TCF4, RCN1, SALL1, MLC1, PSAT1, FAM49A*	*LITAF, SOX9, ADGRV1, MEGF10, NOTCH2, PLCE1, SALL1, BMP7, GPC4, GRIA3, SH3GL2*
OTX1	*GAD2, BHLHE22, ELAVL4, TCF4, ATF4*	*TCF4, BHLHE22*	*NFIX, HES1, CEBPZ*
PAX6	*ELAVL4, NFIB, BHLHE22, SALL1, HOPX*	*ZEB2, NFIB, SEL1L3, BHLHE22, SALL1, FAM49A,*	*NDP, PLCE1, GINS2, TYMS, CNN3, SH3GL2*

		HOPX	
PREB	-	-	*SRRM2, SAMD8, ATP1A3*
REST	*NDP, GAD2, SYT4, CELF3, CTNNA2, ATP1A3, BCAN, HOPX, MEGF10, DLX6, TCF4*	*NDP, GAD2, HOPX, MEGF10, CELF3, CTNNA2*	-
RFX4	*ATP1A3, MYT1L, PHACTR1, MYO10, KIF1A, CTNNA2, RFX4, FAM49A, MSN, CDK4, ITGB8, PDIA6, FAM107A, PDCD4, PON2, IQGAP2, STOML2, CXADR, DLX2, DNAJB5, KNTC1, PTTG1, BTG1, SQLE, ATRX*	*MYO10, KIF1A, PDIA6, RFX4, CTNNA2, STOML2, PDCD4, FAM107A, PHACTR1, ITGB8, FAM49A, CXADR, DLX2, KNTC1, IQGAP2, BSG, SQLE, MSMO1, CDK4*	*SND1, LRIG1, RCN1, PSAT1, ASNS, PDZRN3, PON2, DCXR, MLC1, BMP7, S1PR1, BCHE, SCRG1, PLPP3, NKAIN4, SLC1A3*
SALL1	-	-	*LITAF, MEGF10, NOTCH2, NR2E1, MYO10, PSME1, HAT1, COL4A5, SH3GL2*
SOX2	*RFX4, SRRM4, SALL1, SLC1A2, DNAJB5*	*RFX4, SALL1, SLC1A2*	*PHGDH, MYT1L, TIMP1, LGALS3BP, MPV17, RHOC, FAM49A*
SOX9	*NEUROD2, GAD2, MEGF10, CELF3, SALL1, SLC1A2, DLX6, PPP2R2B, DLX5, RFX4*	*NEUROD2, SALL1, PPP2R2B, SLC1A2, CELF3*	*LTBP1, NOTCH2, NR2E1, BMP7, TNC*
ST18	-	-	*YTHDC1, PDCD4, ARF1, PDZRN3, SEZ6L2, CDK6, SPINT2, CLIP3, ATP6V0B*
STAT1	*FEZF2*	*FEZF2*	*ZNF704, RNASEH2A, HOPX, KLHL28, PON2, PCNA, SAMD8, S1PR1, MCM2*
STAT3	*CTNNA1, LIX1*	*LIX1, CTNNA1*	*MGST1, MCM7, RNASEH2A, SEMA4D, NPTXR, CTNNA2, PCNA, MLC1, FEZF2, GRIA3*

TBR1	*ZEB2*	-	*MGST1, NRXN3, ATP1A2, SLC9A3R1, LRIG1, CXADR, HSDL2, TPP1, KLHL28, ETFB, PON2, MLC1, CTSA, NINJ1, NKAIN4, HMGCS1*
TP53	*ZNF608*	-	-
XBP1	*NR2E1, SOX2, PAX6, CTNNA1, TMEM59L, NFIX, NKAIN4, TUBB2B*	*NR2E1, PAX6, SOX2, CTNNA1, TUBB2B, CREB5*	-
ZBTB18	*CELF3, SOX2, DOK6, NRXN3, LTBP1, CHGB, ZEB2, NRCAM*	*SOX2, NRXN3, LTBP1*	-
ZEB1	*NHLH1, KLHDC8A, LRRN1*	*KLHDC8A, NHLH1, LRRN1, SRP68*	*SFRP1, SLC37A4, NPTXR, NUP107, CDO1, PDE2A, NMT1, DNAJB5, TSPAN6, FDFT1, ID4*

TF	*targets Marbach neurons post-capture*	*targets Marbach astrocytes post-capture*	*targets BrainSpan, BrainCloud post-capture*
AEBP1	-	-	*TIMP1, DNAJB5*
ARID5A	*SALL1*	*SALL1*	-
ARX	*SP8, TBR1, EOMES, GPM6A, ZIC5, ZIC2, ZBTB20, GPM6B, FABP7, ASPH*	*SP8, EOMES, TBR1, GPM6A, ZIC5, SEL1L3, ZIC2, ZBTB20, GPM6B, NRCAM*	*FAM49A, PDPN*
BACH1	*BCL11B, BCL11A, BTBD11, GRHPR, DNAJB5, MPV17, GDAP1L1, ENO2, GLIS3*	*GFAP, DNAJB5, GLIS3, BTBD11, MPV17*	*ZNF704, SMC4, TUBB4A, GFAP, IQGAP2*
BBX	*BCL11B*	*CREB5, NRCAM*	*PDE1C, GNPDA1, SLC25A1*
BCL11B	-	-	*MGST1, MCM7, RNASEH2A, SFRP1, MYT1, NUSAP1, NPTXR, HSDL2, SMC4, ITM2C,*

			NES, STAT3, FEZF2, PGM1, BCL11A, GDAP1L1, SERPINE2, MCM2, ZNF217, POU3F3, AIF1L, GRIA3, DOK5
BHLHE22	*ZBTB20*	*ZBTB20*	*KDM5B, MN1, CXADR, KLF12, SLC6A15, IFITM2, MTSS1, GRHPR, LGALS3BP, GFAP, DDIT3, FAM126A, CTSA, PGM1, BCL11A, TECR, DLX2, DOK5*
BHLHE40	*ZIC1*	*ZIC1*	*MGST1, ATF4, SEMA4D, PAFAH1B3, FEZF2, NEK6, GDAP1L1*
BTG2	-	-	*TOB1, LITAF, GFPT1*
CEBPG	*SALL1, NTRK2, GNG3, NRCAM, BCL11A, HEY1, GPM6B*	*SALL1, NRCAM, NTRK2, GPM6B*	*GLMP, GAD2*
CEBPZ	-	-	*GNG3, ZNF433, ARL6IP6, OTX1*
CREB5	*SOX2, CTNNA1, NFIX, SOX6, DLX6, NKAIN4, BCAN, ZNF711, TMEM59L*	*SOX2, DLX6, CTNNA1, NFIX, ZNF711, CREB5*	*TP53I13, KCNH8, ARF1, TMEM98, CDC42*
DDIT3	-	-	*KDM5B, SLC1A2, SRP68, BHLHE22, CENPF, B9D1, DCC, CXADR, SLC6A15, NPTXR, PDCD4, NCSTN, ETFB, CD9, PPT1, LGALS3BP, CDO1, VPS52, CTSA, BCL11A, NINJ1, RHOC, DOK5*
DLX2	*FEZF2, EMX2, KLHDC8A, ERBB4, GPM6A, SALL1, NRCAM, ELAVL4, LIX1, NTRK3, COL4A6*	*FEZF2, NRCAM, KLHDC8A, MAPK10, THRA, LIX1, SALL1, ERBB4, GPM6A, COL4A6, NR1D1*	*MGST1, ZNF704, ADGRG1, RNASEH2A, BHLHE22, SLC9A3R1, MN1, CXADR, SLC6A15, ATAT1, IFITM2, DUT, BTG1, GRN, LGALS3BP, GFAP, AKAP9, NR1H2, SNCB, TOP2B, VPS52, TMEM35A, PGM1,*

			GDAP1L1, DLX1, NINJ1, SCRG1, SP8, TSPAN6, DOK5
DLX6	EMX2, KLHDC8A, SALL1, NTRK3	KLHDC8A, THRA, SALL1	-
DPF1	-	-	AKAP12, SDCBP, LTA4H
E2F2	KIF1A	KIF1A	KDM5B, PLP1, KNTC1, RNASEH2A, CENPF, SFRP1, NUSAP1, DUT, TMSB15A, TUBB4A, HMGA2, CD9, CDCA7, PPT1, GLI3, GRN, ZNF433, ITM2C, AKAP9, SNCB, IQGAP2, ARL6IP6, MCM2, POU3F3, EOMES, ID4
E2F8	-	OTX2	NRXN1, ASPH, CDKN2D
EBF1	LHX2, SCRT2, DOK6, SLC1A2, NEUROG2, ZBTB20, BCAN, C1orf61	THRA, LHX2, NRXN3, ZBTB20	GFAP
EMX1	FEZF2, NRXN3, MYT1, GPM6B, LIX1, EMX2, ZBTB20, ELMO1	FEZF2, NRXN3, GPM6B, LIX1, EMX2, ELMO1	-
EMX2	FEZF2, CREB5, MYT1, ZBTB20, NRXN3, LIX1, GPM6B, EMX2, KLF7, NFIX	FEZF2, NRXN3, SEL1L3, LIX1, GPM6B, EMX2, ZBTB20, MYT1, NFIX	-
EOMES	GPM6A, NRCAM, NRXN3	GPM6A, NRCAM	KDM5B, PLP1, CENPF, SFRP1, MYT1, NUSAP1, SLC6A15, MTSS1, SCRT2, SMC4, CDCA7, GLI3, E2F2, COL4A6, NES, MLC1, GAD2, IQGAP2, ZNF217, POU3F3, SCRG1, SP8, TSPAN6
FEZF2	-	-	MGST1, RNASEH2A, SEMA4D, RAI14, EMX1, BCL11B, HIST1H4C, PCNA, STAT3,

			BHLHE40, NEUROG2
FOXJ2	*VSTM2A, GPM6A, ZIC1, KLHDC8A, OTX2, BCL11B, SOX6, FAM49A, NFIX, TNC, ELMO1, MAPK10, NTRK2, RFX4*	*THRA, OTX2, ZIC1, ZIC2, DCAF11, KLHDC8A, NFIX, ZIC5, SOX6, BBX*	-
GLI3	-	-	*CENPF, SFRP1, NUSAP1, SMC4, CDCA7, E2F2, COL4A6, AKAP9, ARL6IP6, ZNF217, EOMES, SP8*
GLIS3	-	-	*WDR34*
HBP1	*GPM6A, FAT1, PDE1C*	*GPM6A, FAT1*	-
HES1	-	-	*LTBP1, EMX2, PLCE1, RAI14, GMNN, MAPK10, GPC4, CNN3, TNC, OTX1, PDPN*
HES4	-	-	*PEPD, YIF1A, TUBA1B*
HEY2	*NFIX*	-	*TOB1, HOPX, PSAT1, S1PR1, ALDH9A1, SLC1A3*
HMGA1	*NR2E1, MYT1L*	*NR2E1*	*HOPX, ID3*
HMGA2	-	-	*BTBD11, NUSAP1, GINS2, TGIF1, RAI14, E2F2, ITM2C, SNCB, VPS52, SPINT2, GAD2, SLC6A1*
HSF1	*VSTM2A, HOPX, OTX2, SOX6, ELAVL4, ASPH, CTNNA1, CRYAB, RFX4, PPP2R2B, SRRM4, ZIC1, FAT1*	*OTX2, HOPX, SOX6, CRYAB, ZIC1, FAT1*	-
IER2	-	-	*NRP2, ZWINT*
IRF3	*SP8, OTX2, NRCAM, TCF7L2,*	*SP8, OTX2, NRCAM, NFIX*	*CENPF, MN1, TUBB4A, GRN, ABHD4,*

	CD200, NFIX, AMPH		LGALS3BP, GFAP, CPXM1, AKAP9, SNCB, ATRX, TMEM35A, ATP1B2, IQGAP2, GLIPR2, BSG
KLF12	NSMF, CELF3, ERBB4, ZBTB20, TSPAN3, NR1H2, STAG2, BMP7, VAX1, PAFAH1B3, KIT, HEY2	NSMF, ZBTB20, BMP7, CELF3, KIT, PAFAH1B3	BHLHE22, CRYAB, CCDC88A, SLC6A15, DUT, SMC4, BTG1, GFAP, IFI6, SNCB, FAM126A, SMARCC2, PHF6, PGM1, POU3F3, CCNL1, BSG
LEF1	TBR1, LEF1, ARX, DLX5, DLX6, RFX4, NRXN1, SOX2, SOX6, KCNH8, ZIC2, TUBB4A, THRB, NFIX, NELL2, HEY1, MT3, BTG1, DACH1	MEST, TBR1, ARX, ZIC2, ZIC5, RFX4, NRXN1, SOX2, PIM3, DLX5, KCNH8, LEF1, HMGA2, DACH1	YTHDC1, SLC6A15, MTSS1, BTG1, NES, CCNL1
LHX2	GAD2, TBR1, NRXN3, LIX1, GPM6B, EMX2, BCL11B, ASPH, NHLH1, SALL1, ELMO1	TBR1, NRXN3, SEL1L3, GPM6B, LIX1, EMX2, SALL1	TP53I11, FABP7, STOML2, SLC25A5
LITAF	-	-	RNASEH2A, RAB6A, LTBP1, TP53I13, NRXN1, ADGRV1, NOTCH2, NR2E1, THRA, GNG5, BTG2, SALL1, PSME1, PTBP1, ECI2, PAICS
MAFF	OTX2, BHLHE22, LHX2, TUBB4A	OTX2, BHLHE22, LHX2	GTPBP4
MAFG	OTX2, BHLHE22, TUBB4A, PDE1C	OTX2, BHLHE22	ELMO1, NUP107
MEIS1	FEZF2, GFAP, GPM6A, DNAJB5, ERBB4, BTBD17, NLGN1	FEZF2, NLGN1, GFAP, DNAJB5, GPM6A	STAG2, TMEM98
MN1	-	-	KDM5B, PLP1, SLC1A2, BHLHE22, PEPD,

			SLC6A15, NPTXR, HSDL2, MTSS1, DUT, TMSB15A, HDAC2, ELAVL4, LGALS3BP, GFAP, ITM2C, AKAP9, SNCB, FAM126A, TOP2B, CTSA, SPINT2, PHF6, PGM1, BCL11A, NINJ1, DLX2, IRF3, DOK5
MSX1	*SP8, EOMES, TBR1, DLX6, GPM6A, DLX5, PHF6, ZIC5, HOPX, ZBTB20, BHLHE22, SOX2, ASPH, ZIC2, GPM6B, DLX1, BCL11B, EMX2, DLX2, FAM49A*	*EOMES, SP8, GPM6A, TBR1, HOPX, ZBTB20, ZIC5, GPM6B, ZIC2, SOX2, BHLHE22*	-
MYBL2	*MLC1, NDP*	*NDP, MLC1*	*SLC9A3R1, KCNH8, MTSS1, PKIA, AIF1L, SLC1A3, DOK5*
MYT1	-	-	*MCM7, CAP2, SFRP1, H2AFX, SMC4, BCL11B, NES, TMEM98, SERPINE2, MCM2, POU3F3, EOMES*
MYT1L	-	-	*RREB1, PHGDH, SLC9A3R1, LRIG1, CXADR, TIMP1, SOX2, HSDL2, NPC2, PKIA, ASPH, GRHPR, MLC1, PDE1A, DPYSL2, NINJ1, NELL2, AIF1L, NKAIN4, DOK5*
NEUROD2	*ZBTB20, ZBTB18, GLIS3*	*ZBTB20, ZBTB18*	*ATAT1, MTSS1, SCRT2, GNG3, SNAP25, RAI14, BTG1, PPT1, MAPK10, NES, SNCB, BCL11A*
NEUROG2	*ZBTB20*	*ZBTB20*	*MGST1, RNASEH2A, SEMA4D, NUSAP1, MSMO1, FABP7, DUT, NUP107, TMSB15A, TPP1, CD9, CDCA7, GRN, AKAP9, FAM126A, PAFAH1B3, FEZF2, TMEM35A, SPINT2,*

			PHF6, NEK6, GDAP1L1, NINJ1, DCLK2, POU3F3, NELL2, SP8, TSPAN6, FDFT1
NFE2L2	*BTBD17, GNG3, NTRK2, CRYAB, MAPK10, ZBTB20, HSP90AB1, GPX3, PLCE1, PLBD2, CA12, THSD7A*	*NTRK2, CRYAB, PLCE1, GNG3, CA12, NRXN3, UBC*	*TOB1, PSAT1, PON2, ANP32E, S1PR1*
NFIC	*NEUROD2, RCN1, PPP2R2B, FAM49A, SALL1, FABP7, NHLH1, PSAT1, MYO10, CHD2, MLC1, NDP, NTRK2*	*THRA, RCN1, PPP2R2B, SALL1, PSAT1, HMGA2, MLC1, CHD2*	*TMSB15A, PKIA, AIF1L*
NFIX	-	-	*OTX1, ANKRD36C*
NHLH1	*NES, DNAJB5, BCAN, SOX2, SLC1A2, FAT1, PPP2R5B, SLC9A3R1, STAT3, TNC, ID3, PDPN*	*NES, DNAJB5, FAT1, BCAN, SOX2, SEL1L3, SLC1A2, TNC, LTBP1, STAT3, PDPN*	*GINS2, TYMS*
NPAS3	-	-	*SAE1*
NR1H2	-	-	*ADGRG1, SLC9A3R1, B9D1, ZNF711, ZNF517, CXADR, C1orf61, SLC6A15, IFITM2, DUT, TPP1, PKIA, LGALS3BP, GFAP, IFI6, MPV17, FAM126A, PAFAH1B3, CTSA, PGM1, BCKDK, TECR, VAX2, NINJ1, DLX2, AIF1L, DOK5, BSG*
NR2E1	*NEUROD2, NR2E1, RCN1, FAM49A, SALL1, DLX1, NLGN1, SEL1L3, MLC1, PSAT1, ASPH, DLX2*	*THRA, NR2E1, NLGN1, RCN1, SALL1, MLC1, HMGA2, PSAT1, FAM49A*	*LITAF, SOX9, EMX2, ADGRV1, MEGF10, NOTCH2, PLCE1, ACAA2, SALL1, BMP7, GPC4, GRIA3, SH3GL2*

NR2F2	*KLHDC8A, ID3*	*KLHDC8A*	-
NR4A3	-	-	*DOK6*
OTX1	*GAD2, BHLHE22, ELAVL4, ATF4*	*BHLHE22*	*TRMT112, EMX2, NFIX, HES1, CEBPZ*
OTX2	*BHLHE22, NELL2, ELAVL4, BTG1, PON2*	*ZIC2, BHLHE22, BTG1, NELL2*	*RREB1, PEPD, SOX2, TMEM147*
PBX1	*SCRT2, ZBTB18, GPM6A, DLX1, NDP, DLX2, BHLHE22, NRCAM*	*NRCAM, GPM6A, ZBTB18, SCRT2, NDP, HES1, BHLHE22*	-
PBX3	-	-	*TUBB6*
PHF6	-	-	*MN1, NUSAP1, KLF12, NPTXR, DUT, NUP107, TMSB15A, SMC4, ELAVL4, BTG1, CDCA7, ATP6V0E2, ZNF433, GFAP, AKAP9, SNCB, ENO2, SPINT2, PGM1, NEUROG2, ATP6V0B, DOK5*
POU3F3	*FEZF2, NHLH1, GPM6A, HIVEP3, NEUROG2, RFX4, SCRT2, SOX6, NRCAM, ZBTB18, NRXN3, SLC1A2, NPAS3, ZBTB20, NLGN1, ZIC1, GPM6B, DLX1, MN1, HDAC9, GFAP*	*FEZF2, HIVEP3, NEUROG2, NLGN1, NHLH1, NRCAM, NRXN3, RFX4, ZIC1, SOX6, ZBTB20, GPM6B, NPAS3, SLC1A2, DLX1, ZBTB18, SEL1L3, DACH1, LGALS3BP*	*ZNF704, CAP2, RNASEH2A, SFRP1, SEMA4D, MYT1, NUSAP1, KLF12, NPTXR, NUP107, SMC4, LGMN, TPP1, BTG1, E2F2, BCL11B, NES, SNCB, FAM126A, CTSA, NEK6, PGM1, MCM2, EOMES, NEUROG2*
PREB	-	-	*ATP1A3*
REL	*GPM6A, BHLHE22, RFX4, ZEB1, TNC, DARS*	*GPM6A, BHLHE22, RFX4*	-
REST	*NDP, GAD2, CELF3, NRXN1,*	*NDP, GAD2, NEUROG2,*	*C1orf61*

	PDE1C, NEUROG2, ATP1A3, BCAN, HOPX, MEGF10, DLX6	*HOPX, MEGF10, CELF3, PDE1C*	
RFX4	*ATP1A3, MYT1, MYT1L, ELMO1, MYO10, KIF1A, RFX4, FAM49A, ZIC2, MSN, CDK4, ITGB8, FAT1, FAM107A, PDCD4, PON2, ID2, IQGAP2, STOML2, CXADR, NFIC, DLX2, PDE1C, CCNL1, DNAJB5, KNTC1, NR2F2, MN1, PTTG1, BTG1, SQLE, ATRX*	*ZIC2, ZIC5, MYO10, KIF1A, RFX4, FAT1, STOML2, PDCD4, FAM107A, ID2, ITGB8, ELMO1, FAM49A, CXADR, DLX2, KNTC1, E2F8, IQGAP2, NR2F2, MN1, BSG, SQLE, TECR, MSMO1, CDK4*	*LRIG1, RCN1, PSAT1, PDZRN3, PON2, DCXR, MLC1, BMP7, S1PR1, ALDH9A1, SCRG1, PLPP3, NKAIN4, SLC1A3*
RORA	*NEUROD2, SLC1A3, ELAVL4, BCL11B, CREB5, DACH1, ERBB4, GFAP, HDAC9, DNAJB5, KLHDC8A, PDCD4*	*SLC1A3, NEUROD2, GFAP, DACH1, HDAC9, ZBTB20*	*BTBD11, NRXN3, LRIG1, NARF, GAD2, SERPINE2, SLC6A1, GPM6B, GAD1*
RREB1	*NHLH1, CTNNA1, GAD2, ARX, SCRT2, TBR1, EMX2, OTX1, DCC, ERBB4, FEZF2, OTX2, LRRC3B, BHLHE22, RFX4, SRRM4, COL11A1, EMX1, VAX1*	*OTX2, ARX, EMX2, CTNNA1, FEZF2, NHLH1, CCNI, SEL1L3, LRRC3B, RFX4, TBR1, BHLHE22*	*MYT1L, OTX2*
SALL1	-	-	*LITAF, MEGF10, NOTCH2, NR2E1, MYO10, LTA4H, PSME1, HAT1, COL4A5, SH3GL2*
SCRT2	*SOX2*	*SOX2*	*SLC1A2, ATP1A2, SFRP1, ATAT1, TPP1, SRRM4, RAI14, CD9, ITM2C, IFI6, NEUROD2, NES, NRP2, GAD2, SLC6A1,*

			AIF1L, SCRG1, EOMES, CLIP3, DAP, BSG
SMAD5	-	-	*SFRP1, PLCE1, CDKN2D, GMNN, MAPK10, TMEM98, GPC4, ARL6IP6*
SOX1	*EOMES, OTX2, DLX1, VAX1*	*OTX2, EOMES, SEL1L3, VAX1*	-
SOX2	*RFX4, SRRM4, SP8, SALL1, SOX6, SLC1A2, DNAJB5, NRXN1*	*RFX4, SP8, SALL1, SLC1A2*	*ADGRG1, PHGDH, MYT1L, TIMP1, LGALS3BP, MPV17, RHOC, FAM49A, OTX2*
SOX9	*C1orf61, NEUROD2, GAD2, MEGF10, CELF3, SALL1, SLC1A2, OTX2, DLX6, PPP2R2B, DLX5, RFX4, HDAC9*	*C1orf61, OTX2, NEUROD2, SALL1, PPP2R2B, SLC1A2, CELF3*	*LTBP1, NOTCH2, NR2E1, BMP7, TNC, PAICS*
SP4	*NHLH1, KCNH8, BCL11B, COL11A1, VSTM2A, SRRM4, CELF3, SALL1, RFX4, KLHDC8A, ZIC2, DCC, ZBTB20, SLC1A2, ATP1A3, BMP7, ERBB4, TUBB4A, NR1H2, NR2F2, FEZF2, ZIC5, DACH1, NR4A3, NELL2, SNCB, RAB6A, CDKN1A, SH3GL2, ID4, NCSTN, REL, NTRK2, COL4A6, ISYNA1, KIT, PAFAH1B3, HEY2, FTL, GGNBP2, IQGAP2, TUBB2B, KLF12, MAPK10, DPYSL2, THRB, SP8, ID2, ATP1B2,*	*COL11A1, NHLH1, ZIC2, ZBTB20, SALL1, KLHDC8A, RFX4, ZIC5, NR2F2, FEZF2, DACH1, BMP7, KCNH8, CREB5, NR4A3, NCSTN, CLIC1, DCC, KIT, CELF3, COL4A6, TMEM59L, ID4, ISYNA1, SRRM4, PAFAH1B3, FTL, ID2, INSIG1, RAB6A, CDKN1A, SLC1A2, MAPK10, SNCB, TUBB2B, HMGA1, MN1, PPP2R5B, DPYSL2, TCF7L2, PEPD, ATP1B2, HEY2, MAFF, GLIS3, IER2, MTCH2, TRMT112, GGNBP2,*	*ZNF711, DCC, KLHL28, DCXR, CTSA*

	CA12, CDKN2D, NES, LIG1, TCF7L2, HMGN2, POLR2G, MN1, NPAS3, ZC2HC1A, GLIS3, GAS2L3, ABHD4, ACAA2, C1orf61, CYP51A1, HSDL2	*GAS2L3, GPM6B, SP8, HMGN2, NES, CDK4, SLC9A3R1, ABHD4, ID3, BCL11B, NOTCH2, SH3GL2, CYP51A1, DCAF11, ACAA2, NTRK2, POLR2G, TECR, KNTC1, ERBB4, BTBD11, FAM126A, MPV17, BCKDK, LIG1*	
SP8	*NHLH1, DCC, OTX2, ARX, RFX4, CELF3, BCL11B, COL11A1, NLGN1, KCNH8, KIT, ZBTB20, ERBB4, GNG3, GPM6A, GPM6B, COL4A6, PCLO, VAX1, NPAS3, RORA*	*OTX2, ARX, ZBTB20, COL11A1, RFX4, DCC, GPM6A, GPM6B, NR2F2, KIT, COL4A6, CELF3, GNG3*	*SLC1A2, CENPF, MTSS1, BTG1, CD9, GLI3, GRN, AKAP9, CDO1, PAFAH1B3, GAD2, NINJ1, DLX2, SCRG1, EOMES, NEUROG2, TSPAN6, FDFT1*
ST18	-	-	*YTHDC1, PDCD4, ARF1, PDZRN3, SEZ6L2, SPINT2, CLIP3, ATP6V0B*
STAT3	*CTNNA1, ELMO1, LIX1, HEY1*	*ELMO1, LIX1, CTNNA1*	*MGST1, MCM7, RNASEH2A, SEMA4D, NPTXR, BCL11B, PCNA, MLC1, FEZF2, GDAP1L1, GRIA3*
TBR1	*GPM6A, SOX6*	*GPM6A*	*MGST1, NRXN3, ATP1A2, SLC9A3R1, LRIG1, CXADR, HSDL2, TPP1, KLHL28, ETFB, PON2, MLC1, CTSA, GDAP1L1, NINJ1, NKAIN4, HMGCS1*
TCF7L1	*H2AFX, NRXN1, SOX6, ZIC2, TUBB4A, PDE1C, NRCAM, BTG1, GRIA3, GPM6A,*	*ZIC2, ZIC5, NRCAM, NRXN1, NCSTN, GRIA3, BTG1*	*GMNN*

	IFRD1, NCSTN		
TCF7L2	*ARX, DLX5, NRXN1, DLX6, SOX2, ZIC2, ARID5A, TUBB4A, IFRD1, NRCAM, MYT1, NRXN3, NELL2, GPM6B, PSME1*	*ZIC2, MEST, ZIC5, ARX, NRXN1, SOX2, NRXN3, GPM6B, DLX5*	*RCN1, NOTCH2, NARF*
TEAD2	-	-	*BEX2, PPP2R5B, GFAP*
TGIF1	*GPM6B, BTBD17, DNAJB5, CPE, PTN*	*GPM6B, HEY2, DNAJB5*	*NUSAP1, HMGA2, PLCE1, VIM, ZNF433, LTA4H, MAPK10, CNN3, PTBP1, TUBB6*
THRA	-	-	*RAB6A, LITAF, CDKN2D, PTBP1*
THRB	-	-	*KCNH8, PCLO, TMEM98, ZWINT, DAP*
TP53	*KCNH8*	-	-
VAX1	*SP8, FEZF2, TBR1, NRXN3, LIX1, ERBB4*	*FEZF2, SP8, NRXN3, TBR1, LIX1, LGALS3BP, ZBTB20, ERBB4*	-
VAX2	*SP8, ZIC5, ZIC2, ELAVL4, GPM6B, PPP2R2B, RORA*	*SP8, SEL1L3, ZIC5, ZIC2, GPM6B, ELAVL4, RORA*	*CXADR, MSMO1, PKIA, TUBA1B, NR1H2, PAFAH1B3, NINJ1, AIF1L, MCUB, DOK5*
XBP1	*NR2E1, SOX2, CTNNA1, TMEM59L, NFIX, NKAIN4, TUBB2B, GPM6B*	*NR2E1, SOX2, CTNNA1, TUBB2B, GPM6B, CREB5*	-
ZBTB18	*CELF3, SLC6A1, SOX2, DOK6, NRXN3, LTBP1, CPXM1, NRCAM, ID2*	*SOX2, NRXN3, ID2, LTBP1*	-
ZBTB20	-	-	*MCM7, SFRP1, YTHDC1, TMEM98, ZNF217*
ZEB1	*NHLH1, KLHDC8A, GPM6B, LRRN1*	*KLHDC8A, NHLH1, GPM6B, LRRN1, SRP68*	*SFRP1, NPTXR, NUP107, CDO1, NMT1, DNAJB5, TSPAN6, FDFT1, TPGS2, ID4*

ZIC1	*SCRT2, ELMO1, DLX5, ZIC5*	*DLX5, TNC*	*ZIC2*
ZIC2	-	-	*ZIC1*
ZNF217	-	-	*MCM7, B9D1, SFRP1, YTHDC1, NUSAP1, SLC6A15, HSDL2, RAI14, GLI3, BCL11B, ZBTB20, IQGAP2, BCL11A, MCM2, AIF1L, EOMES, ATP6V0B*
ZNF423	*FEZF2*	*FEZF2*	-
ZNF433	-	-	*KNTC1, TGIF1, E2F2, PHF6, CEBPZ*
ZNF517	-	-	*CENPF, DCC, NUSAP1, CPXM1, AKAP9, NR1H2, VPS52, PGM1*
ZNF711	-	-	*SLC1A2, ZNF704, ATP1A2, RNASEH2A, SLC9A3R1, RBM25, DCC, ATAT1, DUT, BTG1, SP4, AKAP9, NR1H2, FAM126A, CDO1, MLC1, PAFAH1B3, ATP1B2, NEK6, PGM1, HMGCS1, DOK5*

Table S2. Top annotated TF PPIs of the cluster subnetworks of GRNs. The Table correspond to Fig. 4e. The enriched genes for each cell group and each TF PPI are shown for pre-captured and post-capture imputed GRNs

TF	Padj pre-capture	PPI genes pre-capture	Padj post-capture	PPI genes post-capture	Cell type
AR	2.67E-01	*ATRX; TP53*	4.51E-02	*ATRX; ARID5A; REL; PHB; TP53*	N2
ATF3	6.52E-02	*HDAC2; TP53*	1.65E-02	*HDAC2; DDIT3; TP53*	N2
BACH1	2.22E-01	*TP53*	8.09E-03	*MAFF; TP53; NFE2L2*	N2
CEBPB	1.79E-01	*MAPK10; TP53*	2.02E-05	*MAPK10; MEIS1; DDIT3; NFIC; BHLHE40; REL; RORA; TP53*	N2
E2F1	4.17E-02	*HDAC2; CHL1; TP53*	2.09E-02	*HDAC2; LEF1; PHB; TP53*	N2
EP300	4.89E-01	*HDAC2; TP53*	1.63E-02	*MN1; HDAC2; DDIT3; LEF1; REL; RORA; TP53; FOSL2*	N2
ESR1	1.79E-01	*YWHAE; TOP2B; HDAC2; YWHAZ; TP53*	3.79E-02	*YWHAE; TOP2B; HDAC2; THRB; SMARCC2; LEF1; ARID5A; PHB; YWHAZ; TP53*	N2
FOS	2.20E-01	*HDAC2; TUBB2B*	2.06E-02	*TUBB2B; HDAC2; DDIT3; FOSL2; NFE2L2*	N2
HNF4G	1.92E-01	*DLX5*	2.04E-02	*EMX1; DLX5*	N2
JUN	4.30E-02	*MAPK10; HDAC2; CHL1; GAD1*	3.21E-03	*MAPK10; HDAC2; DDIT3; GAD1; LEF1; FOSL2; NFE2L2*	N2
JUND	2.46E-01	*MAPK10*	1.70E-03	*MAPK10; DDIT3; FOSL2; NFE2L2*	N2
KLF4	3.98E-02	*HDAC2; TP53*	4.80E-02	*HDAC2; TP53*	N2
MAFK	2.97E-02	*HDAC2; TP53*	2.42E-03	*HDAC2; TP53; NFE2L2*	N2
NFATC1	9.05E-03	*MAPK10; HDAC2; YWHAZ*	2.48E-03	*MAPK10; HDAC2; REL; YWHAZ*	N2
NFE2	3.34E-01	*DLX5*	7.60E-03	*DLX5; MAFF; AEBP1; NFE2L2*	N2
NFKB1	2.52E-01	*HDAC2; TUBB2B*	3.99E-02	*TUBB2B; HDAC2; REL; PLS3;*	N2

				HMGA2	
NR3C1	4.23E-02	*MAPK10; HDAC2; TUBB2B; TP53*	3.26E-02	*MAPK10; TUBB2B; HDAC2; MAFF; TP53*	N2
RARA	2.11E-02	*MAPK10; TOP2B; HDAC2; BTG1*	1.11E-05	*MAPK10; TOP2B; HDAC2; BTG1; THRB; ERBB4; NR1H2; ARID5A; ZNF423*	N2
RCOR1	2.46E-01	*HDAC2*	1.43E-02	*HDAC2; REST; SMARCC2*	N2
RNF2	2.05E-01	*HDAC2; TP53*	4.56E-02	*HDAC2; REST; PHB; TP53*	N2
RXRA	4.92E-01	*MAPK10*	1.64E-03	*MAPK10; THRB; NR1H2; BHLHE40; ARID5A; ZNF423*	N2
SMAD3	1.00E+00	*TP53*	4.26E-02	*SMARCC2; HEY1; NFIC; LEF1; HMGA2; TP53*	N2
SP1	2.59E-01	*HDAC2; TP53*	4.28E-02	*HDAC2; REST; SMARCC2; REL; TP53*	N2
SP3	4.77E-02	*HDAC2; TP53*	1.05E-02	*HDAC2; REST; TP53*	N2
TP53	1.77E-01	*MAPK10; TOP2B; HDAC2; YWHAZ*	8.28E-03	*CDC42; MAPK10; TOP2B; HDAC2; THRB; ERBB4; BHLHE40; PHB; YWHAZ; PAFAH1B3*	N2
YY1	1.73E-01	*HDAC2; TP53*	2.00E-02	*HDAC2; NR1H2; TP53; NFE2L2*	N2
ZBTB7A	3.80E-02	*HDAC2; TP53*	4.59E-02	*HDAC2; TP53*	N2
ATF2	4.65E-09	*AHCY; PCNA; TPM4; H2AFY; GSTP1; ANXA5; H2AFX; RHOC; HSPD1; HADHB; LDHB; GANAB; BSG; CALU; VIM; P4HB; PFN1; CLIC1; CREB5*	5.65E-08	*AHCY; PCNA; TPM4; H2AFY; GSTP1; ANXA5; H2AFX; PHB; RHOC; DDOST; HSPD1; HADHB; BSG; CALU; VIM; P4HB; PFN1; CLIC1; CREB5*	RGC
ESR1	2.30E-03	*XBP1; DUT; GSN; PARP1; H2AFY;*	8.66E-04	*RPN2; TECR; LEF1; PHB; HMGB1;*	RGC

		STAT3; H2AFX; HMGB1; HSPD1; HADHB; PDLIM1; SLC9A3R1; PTBP1; TUBA1B; GANAB; RFX4; HNRNPA2B1; FLNA; PHGDH; RBBP7; P4HB; PFN1; TP53; CLIC1		*HSPD1; PDLIM1; SLC9A3R1; PTBP1; TUBA1B; FLNA; PHGDH; RBBP7; CLIC1; XBP1; DUT; TCF7L1; GSN; PARP1; H2AFY; H2AFX; DDOST; HADHB; RFX4; HNRNPA2B1; P4HB; PFN1; SLC25A5*	
GLI1	3.13E-01	*XBP1; CDK4*	3.59E-02	*XBP1; ZIC2; CDK4; ZIC1*	RGC
MYC	3.09E-04	*SLC25A1; RNH1; MCM7; CDCA7L; IQGAP2; SMC4; SMC2; HSPD1; PTBP1; TUBA1B; TUBB6; GANAB; FLNA; AASS; RRM1; STAT1; H2AFZ; HADHB; RCN1; CDK6; FANCD2; CDK4; PRC1; PPT1; MCM3; MCM4; TPP1; TP53*	1.01E-02	*SLC25A1; RNH1; RPN2; MCM7; CDCA7L; LEF1; IQGAP2; SMC4; SMC2; HSPD1; PTBP1; TUBA1B; TUBB6; FLNA; RRM1; H2AFZ; HADHB; RCN1; STAG2; FANCD2; CDK4; PRC1; PPT1; MCM3; TPP1; NFE2L2*	RGC
NRF1	3.65E-01	*PARP1*	9.22E-03	*PARP1; MAFF; NFE2L2*	RGC
SMAD2	2.70E-02	*TUBA1B; ZEB1; CDK4; FLNA; SOX9; SRI; TP53; LITAF; RHOA; GFAP; HSPD1; SOD1*	3.19E-03	*TGIF1; LEF1; SRI; LITAF; GLI3; RHOA; GFAP; HSPD1; SOD1; TUBA1B; ZEB1; CDK4; ANP32E; FLNA; SOX9; SLC25A5*	RGC
SOX2	2.66E-02	*SALL1; ZEB1; PAX6; RBBP7; SAE1*	3.69E-02	*TCF7L1; SALL1; ZEB1; RBBP7; SAE1*	RGC
SP1	2.40E-02	*TUBA1B; REST; PARP1; TPM4; CDK4; STAT3; S1PR1; NR2E1; VIM; TP53*	3.55E-02	*TUBA1B; BCL11B; PARP1; TPM4; CDK4; SP4; S1PR1; MSX1; NR2E1; VIM*	RGC
TCF3	8.40E-02	*SLC9A3R1; PCNA; CDK4; RFX4; PON2; ID4; ID3; RBBP7*	1.20E-02	*EEF1B2; SLC9A3R1; PCNA; RPN2; CDK4; RFX4; ID2; PON2; ID4; ID3;*	RGC

				RBBP7	
AR	0.088684	*KDM5B; DCC; UBC; TCF4; KIF1A; ENO2; CALM1*	0.048663	*KDM5B; BTG2; DCC; UBC; ARID5A; KIF1A; ENO2; CALM1*	N1
ATF3	0.092944	*HSP90AB1; CEBPG; UBC; ATF4*	0.000486	*HSP90AB1; DDIT3; CEBPG; UBC; HSF1; ATF4*	N1
BACH1	0.155689	*ARF1; UBC*	0.00079	*ARF1; MAFG; UBC; MAFF; NFE2L2*	N1
CEBPB	0.085313	*UBC; CEBPG; POLE3; CAMK2G; ATF4*	0.00297	*MEIS1; DDIT3; UBC; CEBPG; HSF1; RORA; HDAC9; ATF4*	N1
EP300	0.284725	*CDKN1A; NPM1; UBC; HBP1; TCF4; ATF4*	0.04952	*MN1; XBP1; CDKN1A; THRA; IRF3; DDIT3; UBC; HBP1; RORA; ATF4; FOSL2*	N1
ESR1	0.10725	*SRRM2; MTCH2; ARF1; CDKN1A; NPM1; HSP90AB1; ASNS; GNL3; UBC; HNRNPC; KIF1A; CALM1; SPTAN1*	0.049891	*XBP1; ARF1; CDKN1A; MTCH2; HSP90AB1; THRB; THRA; ARID5A; HDAC9; GTPBP4; GNL3; FKBP1A; UBC; COX2; HNRNPC; KIF1A; CALM1*	N1
FOS	0.20152	*NTRK3; UBC; CEBPG; ATF4*	0.015839	*XBP1; DDIT3; NTRK3; UBC; CEBPG; ATF4; FOSL2; NFE2L2*	N1
JUN	0.060426	*CDKN1A; PPP2R2B; NTRK3; UBC; VRK1; AMPH; TCF4; ATF4*	0.001459	*CDKN1A; DACH1; PPP2R2B; DDIT3; NTRK3; UBC; AMPH; HDAC9; ATF4; FOSL2; NFE2L2*	N1
JUNB	0.087772	*NPM1; UBC; ATF4*	0.021055	*MAFG; UBC; FOSL2; ATF4*	N1
JUND	0.411506	*UBC*	0.016146	*DDIT3; UBC; FOSL2; NFE2L2*	N1
LMO2	0.079107	*KDM5B; NHLH1; UBC*	0.015845	*KDM5B; BEX2; NHLH1; UBC*	N1
MAFK	0.285892	*UBC*	0.001669	*NR4A3; UBC; BACH1; NFE2L2*	N1
MEF2A	0.391665	*NFIX*	0.04559	*NFIX; THRA; HDAC9*	N1

NFE2L2	0.10928	*CDKN1A; UBC; ATF4*	0.000282	*CDKN1A; DDIT3; MAFG; UBC; MAFF; BACH1; ATF4*	N1
NRF1	0.216054	*UBC*	0.003623	*MAFF; UBC; NFE2L2*	N1
PPARG	0.188598	*HSP90AB1; UBC; KIF1A*	0.046737	*HSP90AB1; UBC; MAFF; KIF1A; NFE2L2*	N1
RXRA	0.404191	*UBC; KIF1A*	0.0303	*THRB; THRA; NR1H2; UBC; ARID5A; KIF1A*	N1
TCF3	0.074468	*SOX2; NPM1; NHLH1; UBC; TCF4; CALM1; MRPL44*	0.034499	*SOX2; NHLH1; UBC; AEBP1; CALM1; NEUROG2; MRPL44; RAI14*	N1
TP53	0.129674	*CDKN1A; NPM1; HSP90AB1; PPP2R2B; IFRD1; UBC; NMT1; VRK1; CEBPZ; GNL3*	0.036786	*BTG2; CDKN1A; HSP90AB1; THRB; IFRD1; BACH1; HDAC9; GTPBP4; GNL3; PPP2R2B; UBC; NMT1; ANXA7; CEBPZ*	N1
YY1	0.377623	*NPM1; UBC*	0.049365	*FKBP1A; NR1H2; UBC; PHC3; NFE2L2*	N1
ZNF217	0.077087	*KDM5B; UBC*	0.049583	*KDM5B; UBC*	N1

Table S3. Comparison of the significance of TF-based cell type identification in pre- and post-capture datasets. For each cell type, the sets of targeted TF marker DEGs (based on TF clustering of 585 pre- or 731 post-capture TFs, Fig. S3) was compared to the full sets of TF markers in the reference datasets using a hypergeometric test. P values of hypergeometric test show enrichment of the pre- and post-capture DEGs with targeted TF markers.

cell type	pre-capture P value	post-capture P value
neurons	0.015	$1.2 \cdot 10^{-10}$
astrocytes	$< 2.2 \cdot 10^{-16}$	$3.5 \cdot 10^{-7}$
oligodendrocytes	$6.1 \cdot 10^{-4}$	0.0015
endothelial cells	0.019	0.0016

Table S4. Targeted TFs, which are differentially expressed between different cell populations of intestinal stromal cells from UC patients. The analysis was done using Seurat package in R and standard Seurat outputs are shown for pre- and post-capture, as indicated. logFC are averaged natural log fold changes in a gene expression in cluster 1 relative to cluster 0; pct.1 and pct.2 are relative number of cells expressing the gene in clusters 1 and 0, respectively.

	logFC POST	pct.1 POST	pct.2 POST	Padj POST	logFC PRE	pct.1 PRE	pct.2 PRE	Padj PRE
TCF7L2	-0.58	0.92	0.987	0.0038	0.59	0.2	0.471	1
PBX1	-1.74	1	0.96	0.0096	-0.19	0.175	0.612	0.37
TCF21	-2.78	0.84	0.907	0.053	-0.49	0.05	0.306	1
NFATC4	-2.11	0.62	0.84	0.072	-0.42	0.1	0.247	1
FOXO3	-0.71	1	0.987	0.077	-0.72	0.225	0.529	1

References

Colantuoni C, Lipska BK, Ye T, Hyde TM, Tao R, Leek JT, Colantuoni EA, Elkahloun AG, Herman MM, Weinberger DR et al. 2011. Temporal dynamics and genetic control of transcription in the human prefrontal cortex. *Nature* **478**(7370): 519-523.

Curion F, Handel AE, Attar M, Gallone G, Bowden R, Cader MZ, Clark MB. 2020. Targeted RNA sequencing enhances gene expression profiling of ultra-low input samples. *RNA biology*: 1-13.

Inoue F, Kreimer A, Ashuach T, Ahituv N, Yosef N. 2019. Identification and Massively Parallel Characterization of Regulatory Elements Driving Neural Induction. *Cell stem cell* **25**(5): 713-727 e710.

Lachmann A, Giorgi FM, Lopez G, Califano A. 2016. ARACNe-AP: gene network reverse engineering through adaptive partitioning inference of mutual information. *Bioinformatics* **32**(14): 2233-2235.

Marbach D, Lamparter D, Quon G, Kellis M, Kutalik Z, Bergmann S. 2016. Tissue-specific regulatory circuits reveal variable modular perturbations across complex diseases. *Nature methods* **13**(4): 366-370.

Stuart T, Butler A, Hoffman P, Hafemeister C, Papalexi E, Mauck WM, 3rd, Hao Y, Stoeckius M, Smibert P, Satija R. 2019. Comprehensive Integration of Single-Cell Data. *Cell* **177**(7): 1888-1902 e1821.
